# Efficacy and tolerability of the Subcutaneous Semaglutide for type 2 Diabetes patients: an updated systematic review and meta-analysis

**DOI:** 10.1186/s13098-023-01195-7

**Published:** 2023-10-28

**Authors:** Shanshan Hu, Xiaorong Su, Guorong Fan

**Affiliations:** 1 Department of Clinical Pharmacy, Shanghai General Hospital, Shanghai Jiao Tong University School of Medicine, Shanghai, 200080 China; 2 Department of Pharmacy, Xiamen Maternal and Child Health Hospital, Xiamen, 361003 China

**Keywords:** Semaglutide, Type 2 Diabetes, Randomized controlled trials, Meta-analysis

## Abstract

**Objectives:**

To update and assess the efficacy and tolerability of once weekly subcutaneous semaglutide in patients with type 2 diabetes (T2D).

**Materials and methods:**

PubMed, Science Direct, Cochrane Library, Clinical trial, Springer, OVID, China National Knowledge Infrastructure (CNKI), WanFang Data and China Science and Technology Journal Database (VIP) were searched from inception to January 18, 2023. Randomized controlled trials (RCTs) comparing subcutaneous semaglutide with placebo or any other antidiabetic agent in adults with T2D were eligible. The risk ratio (RR) and mean difference (MD) with 95% confidence intervals (CIs) were determined to synthesize the results.

**Results:**

A total of 17 trials enrolling 14,940 T2D patients were included. For efficacy, compared with placebo, semaglutide exhibited beneficial effects on glycosylated hemoglobin A1c (HbA1c) control [MD -0.97%, 95% CI (-1.33, -0.62), *I*^*2*^ = 91%; MD -1.36%, 95% CI (-1.59, -1.13), *I*^*2*^ = 84%, semaglutide 0.5 and 1.0 mg, respectively], body weight reduction, blood pressure control. At the same time, subcutaneous semaglutide 0.5 and 1 mg reduced HbA_1c_ by 0.56% (95% CI 0.32 to 0.80) and 0.63% (95% CI 0.35 to 0.91) compared to other glucose-lowering agents. For tolerability, semaglutide did not increase the incidence of adverse events (AEs) and serious adverse events (SAEs), severe or blood glucose (BG) confirmed hypoglycaemia, acute pancreatitis and diabetic retinopathy compared to placebo or active comparators, but did increase the risk of nausea, diarrhea and vomiting.

**Conclusions:**

Semaglutide has a better effect on glycaemic control and weight loss than other therapies. Nevertheless, semaglutide was associated with increased incidence of gastrointestinal-related disorders. Further large, multicenter randomized controlled clinical trials are still needed to obtain more robust evidence to better guide clinical treatment decisions.

**Supplementary Information:**

The online version contains supplementary material available at 10.1186/s13098-023-01195-7.

## Introduction

Type 2 diabetes (T2D) is a metabolic syndrome characterized by long-term hyperglycemia, which is caused by insulin resistance and/or impaired pancreatic β-cell function [1]. According to the American Diabetes Association (ADA) and European Association of Securities Dealers (ESAD) consensus report 2022, glucagon-like peptide-1 receptor agonists (GLP-1 RAs) are recommended as first-line therapy for people with combined atherosclerotic cardiovascular disease and the high-risk factors for cardiovascular disease (CVD) [2, 3].

Semaglutide is a newly approved GLP-1 RA that reduces glucose levels by improving β-cell response, inhibiting glucagon secretion, and delaying gastric emptying [4, 5]. In addition, semaglutide has direct cardiovascular benefits in patients with T2D and is associated with a low risk of hypoglycemia [6, 7]. Subcutaneous semaglutide was approved for use as an adjunctive therapy to diet and exercise to improve glycemic control for T2D by the United States Food and Drug Administration (FDA) on December 5, 2017.

To date, in addition to the network meta-analysis, four meta-analyses have evaluated the efficacy and tolerability of subcutaneous semaglutide in T2D patients [8–11]. In the intervening 5 years since previous meta-analyses, several new RCTs have been completed to evaluate the efficacy and tolerability of semaglutide. For instance, SUSTAIN 8 was the first head-to-head phase III clinical trial comparing semaglutide and canagliflozin on the basis of metformin [12]. SUSTAIN 9 compared semaglutide to placebo as an add-on to SGLT-2 inhibitor therapy [13]. SUSTAIN 10 was the first head-to-head phase III clinical trial comparing semaglutide to liralutide [14]. SUSTAIN CHINA compared semaglutide to sitagliptin in a predominantly Chinese population [15]. SURPASS 2 was the first head-to-head phase III clinical trial comparing tirzepatide to semaglutide [16].

There are two main aims to update the meta-analysis of subcutaneous semaglutide. Firstly, four meta-analyses have been evaluated the efficacy and tolerability of subcutaneous semaglutide in 2018 [8–11]. Since then, five RCTs [11–15] have been published on the efficacy of subcutaneous semaglutide for T2D patients. However, there is no updated meta-analysis on subcutaneous semaglutide. Secondly, the previous results regarding the efficacy and safety of subcutaneous semaglutide on T2D have not been entirely consistent. For example, SUSTAIN 1–7 studies [17] have provided extensive evidence that semaglutide appeared more effective than other treatments. However, in the SURPASS 2 [16] trial, tirzepatide exhibited a more outstanding potent of hypoglycemic and weight-lowering than semaglutide. There is still a paucity of comprehensive and up-to-date evaluations of the available results that incorporate data from all relevant RCTs published to date. Therefore, an update systematic review and meta-analysis were applied to conclude the efficacy and tolerability of subcutaneous semaglutide in T2D patients, comprehensively and authenticly.

## Methods

This research was operated according to the Preferred Reporting Items for Systematic Reviews and Meta-Analyses (PRISMA) statement [18, 19], which protocol was registered in PROSPERO (CRD42021264640).

### Data sources and search strategies

The following electronic databases were searched: PubMed, Science Direct, Cochrane Library, Clinical trials, Springer, OVID, China National Knowledge Infrastructure (CNKI), WanFang Data, and the China Science and Technology Journal Database (VIP) from inception to January 18, 2023. The selected terms and search combinations were: “semaglutide” or “NN9535” in combination with “Diabetes Mellitus, Type II” or “Diabetes Mellitus, Noninsulin-Dependent” or “T2D”. The detailed search strategy is provided in Table S1.

### Study selection

Two reviewers (Hu and Su) independently identified studies and did screening and data extraction, while any disagreements were resolved by the third reviewer (Fan). RCTs that compared once-weekly subcutaneous semaglutide with placebo or any other antidiabetic agent in adults with T2D were included. Search results were imported into Endnote, Clarivate Analytics, a reference management software, for deduplication. After removing duplication, two reviewers independently screened all records by title and abstract in duplicate. Subsequently, potentially eligible records were assessed in full text.

The detailed inclusion and exclusion criteria were listed in Table S2. For simplicity, the main inclusion criteria were: (1) RCTs that compared subcutaneous semaglutide with placebo or any other active comparator in adults with T2D and HbA_1c_ ≥ 7%; (2) treatment duration ≥ 12 weeks; (3) the primary outcome for this meta-analysis must be reported in the trial: reduction in HbA1c. The main exclusion criteria were: (1) RCTs not for T2D, but obesity, impaired glucose tolerance, gestational diabetes or type1 diabetes; (2) papers published in form of abstracts, review articles, hoc-analysis, pharmacoeconomics research or letter and comments; (3) duplicate studies.

### Data extraction and quality assessment

The extracted information included: first author, publication year, National Clinical Trial (NCT) number, duration of treatment, intervention in each trial arm, sample size, average age, diabetes duration, baseline HbA1c, body weight. The primary outcome for this meta-analysis was the change in HbA1c from baseline. Secondary efficacy outcomes included the change in body weight, fasting plasma glucose (FPG), self-measured plasma glucose (SMPG), systolic blood pressure (SBP), diastolic blood pressure (DBP), the number of participants achieving HbA1c<7%, the proportion of patients achieving weight loss ≥ 5% and ≥ 10%, the number of participants achieving HbA1c<7.0% without severe or BG-confirmed hypoglycaemia and without weight gain. Tolerability outcomes consisted of the incidence of adverse events (AEs), serious AEs (SAEs), hypoglycaemic events (severe or BG-confirmed symptomatic). With additional tolerability outcomes, the incidence of gastrointestinal adverse events (nausea, vomiting, diarrhoea), acute pancreatitis, and diabetic retinopathy were summarized.

The quality of the included studies was assessed using the Cochrane Collaboration’s Risk of Bias Tool [20]. Two reviewers independently assessed the risk of bias for including studies in duplicate, and any discrepancies were resolved by the third reviewer. Overall risk of bias was considered ‘low’ if all domains were rated as low risk of bias, ‘high’ if there is a high risk of bias in at least one domain, and of some concern in any other case.

### Data synthesis

Continuous data were analyzed using mean differences (MD) and 95% confidence intervals (CIs) to express effect size. Dichotomous data were reported using the risk ratios (RRs) and 95% CIs. *P* < 0.05 *(*two*-*tailed*)* was considered significant. Heterogeneity across studies was evaluated by Cochrane’s *Q* and *I*^*2*^ statistics, considering the *P* value less than 0.10 or the inconsistency index (*I*^*2*^) statistic greater than 50% indicative of significant heterogeneity [18]. Pooled analyses were conducted using a random-effects model. In case of considerable heterogeneity (*p*<0.10 and *I²*>50%), sensitivity analysis was performed by excluding each included study one by one and then re-estimating the combined outcomes. The fixed-effects model was used for sensitivity analysis. We performed pooling analyses of different outcomes based on placebo-controlled and active comparator trials. In addition, subgroup analysis was performed according to the different doses of subcutaneous semaglutide (0.5 mg and 1.0 mg) and the type of active comparators. Statistical analysis was performed using Review Manager V.5.4 statistical software.

## Results

### Results of search and study characteristics

The flowchart of the study selection process is shown in Fig S1. A total of 17 trials enrolling 14,940 T2D patients were included in this systematic review and meta-analysis [12–16, 21–32]. Details regarding the characteristics of the included studies and patients at baseline are summarized in Table 1. Among 17 studies, subcutaneous semaglutide was compared with the placebo or with the other antidiabetic agent in 5 studies [13, 24, 27, 30, 32] and 10 studies [14–16, 21–23, 25, 26, 29, 31], respectively, while 2 trials [12, 28] compared both the placebo and the other antidiabetic agent. Regarding the active comparators in included studies, 3 studies received liraglutide [11, 13, 26], two studies received another GLP-1 RA (dulaglutide [27] or exenatide [19]), 3 studies [14, 20, 29] received sitagliptin, 1 study received tirzepatide [15], 1 study received insulin glargine [21] and 1 study received the additional oral antidiabetic drugs [23]. In the add-on trials, insulin, metformin, sulfonylurea, thiazolidinedione, and other oral antidiabetic drugs were used as the background therapy in 15 trials [12–16, 21–31]. Only 1 trial enrolled treatment-navie participants [32].

**Table 1:** Baseline characteristics of the included studies

Trial name	Treatment duration, (weeks)	Backgroud therapy	Study arms	Patients (n)	Age (year)	Body weight (kg)	Diabetes duration (years)	Baseline HbA1c ( %)
Sorli 2017 NCT02054897	30	diet and exercise	Semaglutide 0.5 mg	128	54.6	89.8	4.81	8.1
			Semaglutide 1.0 mg	130	52.7	96.9	3.62	8.1
			placebo	129	53.9	89.1	4.06	8.0
Ahren 2017 NCT01930188	56	MET ± TZD	Semaglutide 0.5 mg	409	54.8	89.9	6.4	8.0
			Semaglutide 1.0 mg	409	56.0	89.2	6.7	8.0
			Sitagliptin 100 mg	407	54.6	89.3	6.6	8.2
Ahmann 2018 NCT01885208	56	MET ± (TZD or SU)	Semaglutide 0.5 mg	404	56.4	96.2	9.0	8.4
			Exenatide 2 mg	405	56.7	95.4	9.4	8.3
Aroda 2017 NCT02128932	30	MET ± SU	Semaglutide 0.5 mg	362	56.5	93.7	7.8	8.1
			Semaglutide 1.0 mg	360	56.7	94.0	9.3	8.3
			Insulin	360	56.2	92.6	8.6	8.1
Rodbard 2018 NCT02305381	30	BI ± MET	Semaglutide 0.5 mg	132	59.1	92.7	12.9	8.4
			Semaglutide 1.0 mg	131	58.5	92.5	13.7	8.3
			placebo	133	58.8	89.9	13.3	8.4
Marso 2016 NCT01720446	104	<2 OADs ± (BI or PRI)	Semaglutide 0.5 mg	826	64.6	91.8	14.3	8.7
			Semaglutide 1.0 mg	822	64.7	92.8	14.1	8.7
			Placebo 0.5 mg	824	64.8	91.8	14.0	8.7
			Placebo 1.0 mg	825	64.4	91.9	13.2	8.7
Partley 2018 NCT02648204	40	MET	Semaglutide 0.5 mg	301	56	96.4	7.7	8.3
			Semaglutide 1.0 mg	299	55	95.5	7.0	8.2
			Dulaglutide 0.5 mg	300	55	95.6	7.3	8.2
			Dulaglutide 1.0 mg	299	56	93.4	7.6	8.2
Lingvay 2019 NCT03136484	52	MET	Semaglutide 1.0 mg	394	55.7	90.6	7.5	8.3
			Canagliflozin 100 mg	394	57.5	89.8	7.2	8.2
Zinman 2019 NCT03086330	30	SGLT-2 inhibitor	Semaglutide 1.0 mg	151	57.5	89.6	9.8	8.0
			placebo	151	56.6	93.8	9.6	8.1
Capehorn 2020	30	MET ± SU ± SGLT-2	Semaglutide 1.0 mg	290	60.1	96.9	9.6	8.2
			Liraglutide 1.2 mg	287	58.9	97.2	8.9	8.3
Nauck 2016 NCT00696657	12	diet and exercise ± MET	placebo	46	55.3	90.5	2.4	8.1
			Semaglutide 0.4 mg	48	53.8	87.0	2.0	8.1
			Semaglutide 0.8 mg	43	55.9	85.7	2.6	8.0
			Liraglutide 1.2 mg	45	54.8	90.5	3.3	8.0
			Liraglutide 1.8 mg	50	54.3	87.2	2.5	8.1
Ji 2020 NCT03061214	30	MET	Semaglutide 0.5 mg	288	53.0	77.6	6.3	8.1
			Semaglutide 1.0 mg	290	53.0	76.1	6.7	8.1
			Sitagliptin 100 mg	290	53.1	75.5	6.1	8.1
Lingvay 2018 NCT02461589	26	diet and exercise ± MET	placebo	129	57.1	94.0	7.1	8.1
			Semaglutide 0.05 mg/d	64	57.5	93.4	6.5	7.9
			Semaglutide 0.1 mg/d	63	58.4	92.4	8.1	7.9
			Liraglutide 1.2 mg	64	53.7	96.7	6.9	8.1
			Liraglutide 1.8 mg	65	55.8	93.4	6.6	8.1
Davies 2017 NCT01923181	26	diet and exercise ± MET	Semaglutide 1.0 mg	71	56.8	88.8	5.6	7.8
			placebo	69	58.9	93.8	6.7	8.0
Seino 2017 NCT02254291	30	diet and exercise or OAD monotherapy	Semaglutide 0.5 mg	103	58.8	67.8	8.0	8.2
			Semaglutide 1.0 mg	102	58.1	70.8	7.8	8.0
			Sitagliptin 100 mg	103	57.9	69.4	8.1	8.2
Kaku 2018 NCT0220737	56	SU or GLI or AGI or TZD	Semaglutide 0.5 mg	239	58.0	71.0	8.1	8.0
			Semaglutide 1.0 mg	241	58.7	71.7	9.4	8.1
			Additional OAD	121	59.2	72.2	9.3	8.1
Frías 2021 NCT03987919	40	MET	5 mg Tirzepatide	461	56.3	925	9.1	8.32
			10 mg Tirzepatide	459	57.2	94.8	8.4	8.3
			15 mg Tirzepatide	464	55.9	93.8	8.7	8.26
			1 mg Semaglutide	461	56.9	93.7	8.3	8.25

Abbreviations: AGI, a-Glycosidase inhibitor; BI, basal insulin; HbA1c, glycated haemoglobin; MET, metformin; OAD, oral antidiabetic drug; PRI, premixed insulin; SGLT-2, sodium-dependent glucose transporters 2; SU, sulfonylurea; TZD, thiazolidinedione

Remarkably, in dose-finding trials, we extracted data from the 0.5 and 1.0 mg arms that were approved by the FDA. 2 studies [12, 28] used subcutaneous semaglutide at a different dose than approved, so similar dose arms were used instead. The treatment duration ranged from 12 to 104 weeks. The 30-week trials are the most common among them. It is worth mentioning that the SUSTAIN 6 study aimed to evaluate the effect of semaglutide treatment on cardiovascular events and other long-term outcomes in patients with T2D, so the study duration was as long as 104 weeks [27]. In addition, the SURPASS 2 study compared the hypoglycemic effect of tirzepatide 5 mg, 10 mg, and 15 mg with that of semaglutide 1 mg, therefore the initial dose of tirzepatide 5 mg was used in this study to compare with semaglutide 1 mg [16].

### Risk of bias assessment

**Fig. 1:**
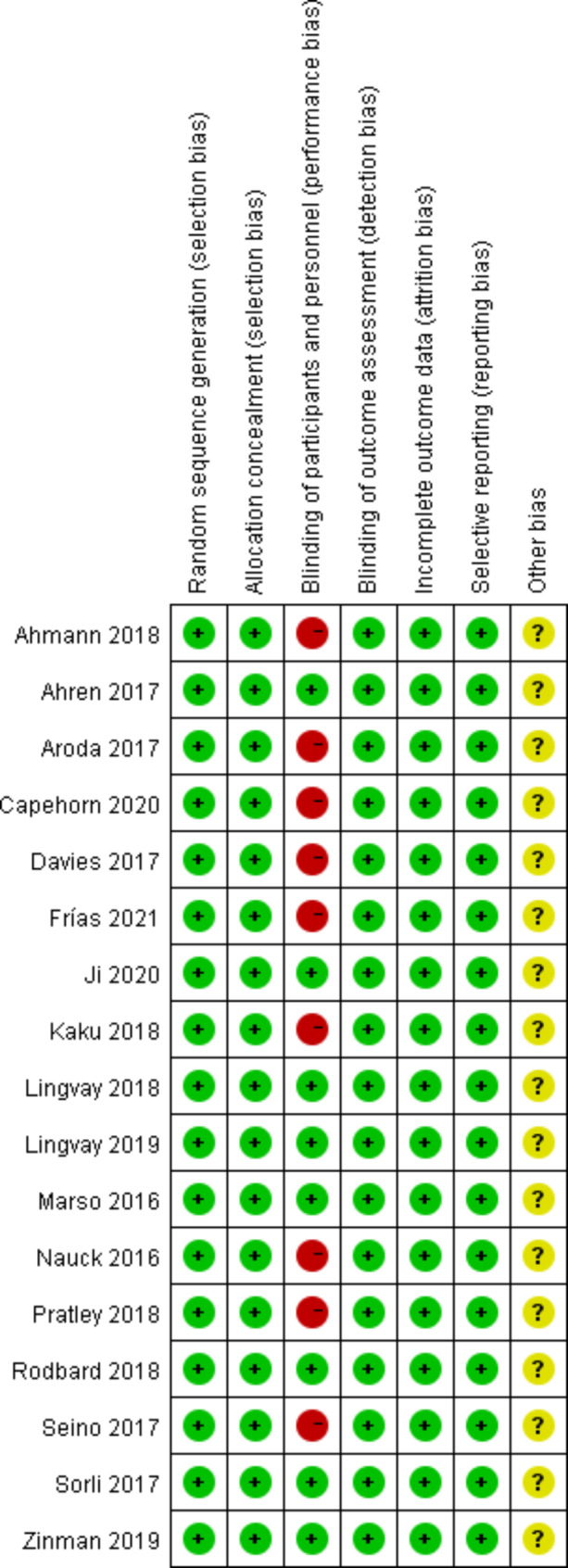
Risk of bias summary: review authors’ judgements about each risk of bias item for each included study

The risk of bias assessment for all trials is presented in Fig. 1. In total, 8 trials [12, 13, 15, 22, 26, 27, 30, 32] were designed as double-blind type, while 9 trials [14, 16, 21, 23–25, 28, 29, 31] were open-label type. Therefore, there was high potential risk in blinding of participants and personnel domains in open-label trials. Other sources of bias were low in all trials.

### Efficacy outcomes

#### Haemoglobin A_1c_

**Fig. 2:**
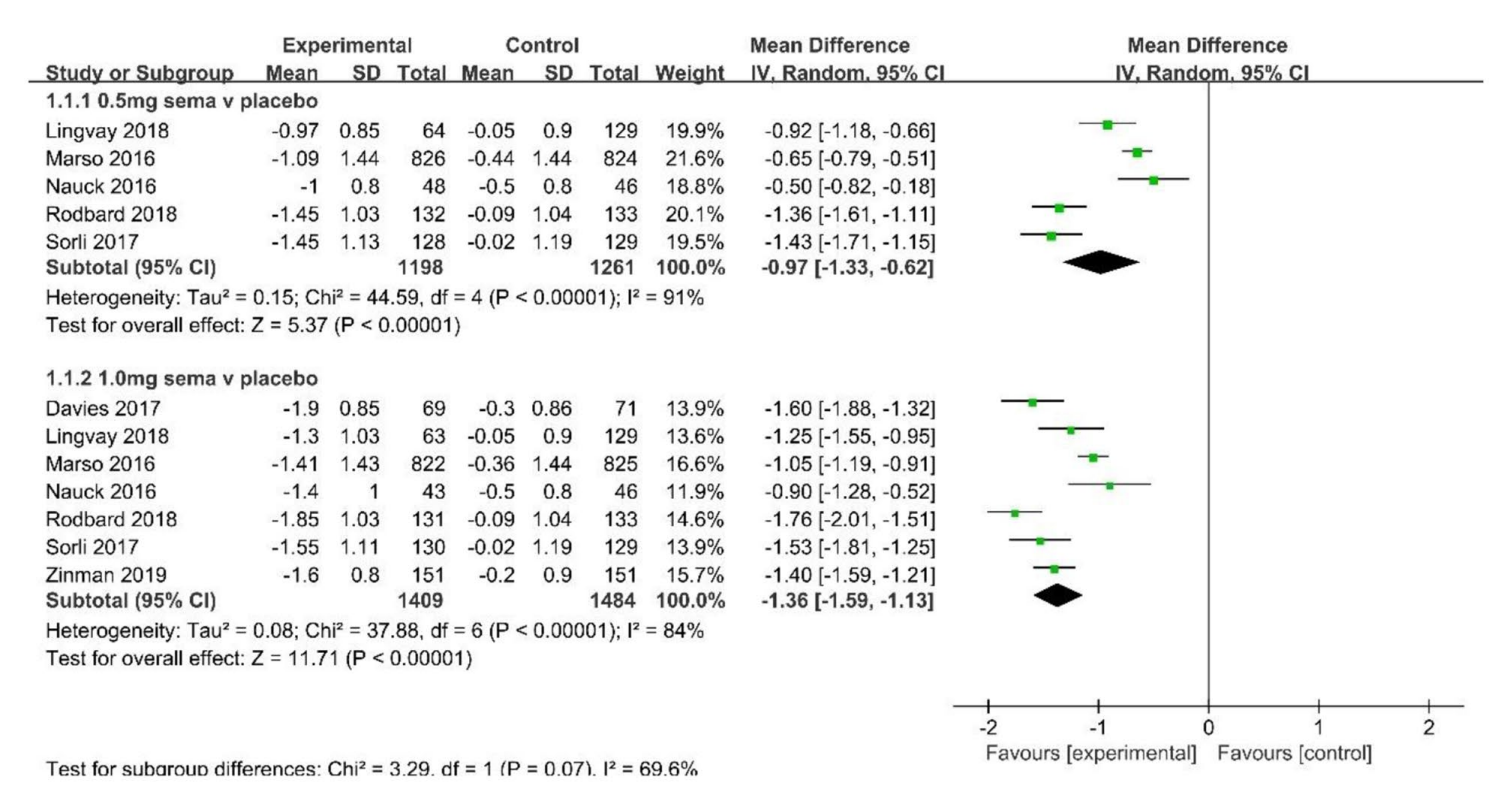
Mean difference of change in HbA1c (%) between semaglutide and placebo

Compared with placebo, semaglutide 0.5 and 1.0 mg reduced HbA1c by 0.97% [95% CI (-1.33, -0.62); *I*^*2*^ = 91%, 5 studies] and by 1.36% [95% CI (-1.59, -1.13); *I*^*2*^ = 84%, 7 studies], respectively (Fig. 2).

**Fig. 3:**
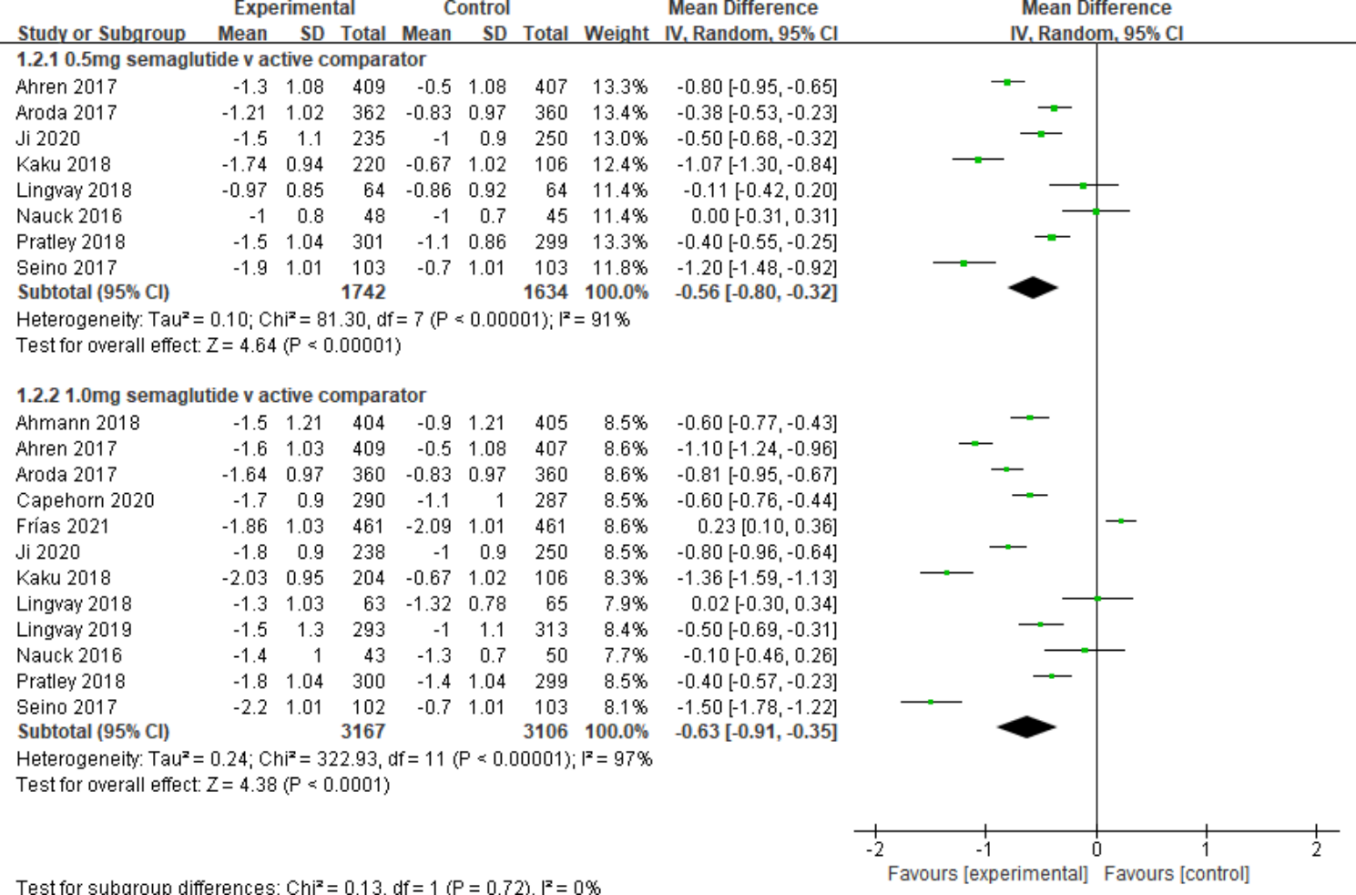
Mean difference of change in HbA1c (%) between semaglutide and active comparator

Compared to other active comparator, semaglutide was linked to a significant reduction in HbA1c [MD -0.56%, 95% CI (-0.80, -0.32); *I*^*2*^ = 91%, 8 studies; MD -0.63%, 95% CI (-0.91, -0.35); *I*^*2*^ = 97%, 12 studies for semaglutide 0.5 and 1.0 mg, respectively] (Fig. 3). Results were consistent both in a sensitivity analysis excluding the study that used lower semaglutide doses [MD -0.71%, 95% CI (-0.96, -0.46); *I*^*2*^ = 91%, 6 studies; MD -0.74%, 95% CI (-1.04, -0.43); *I*^*2*^ = 97%, 10 studies for semaglutide 0.5 and 1.0 mg, respectively] and in a sensitivity analysis including only trials at low risk of bias.

Subgroup analyses performed according to the category of other antidiabetic agents showed that semaglutide was more efficacious compared to GLP-1 RAs. In addition, compared to sitagliptin, semaglutide significantly decreased the level of HbA1c. However, treatment with tirzepatide significantly reduced HbA1c by 0.23% [95%CI (0.10, 0.36)] compared with semaglutide (Table S3).

#### Body weight

**Fig. 4:**
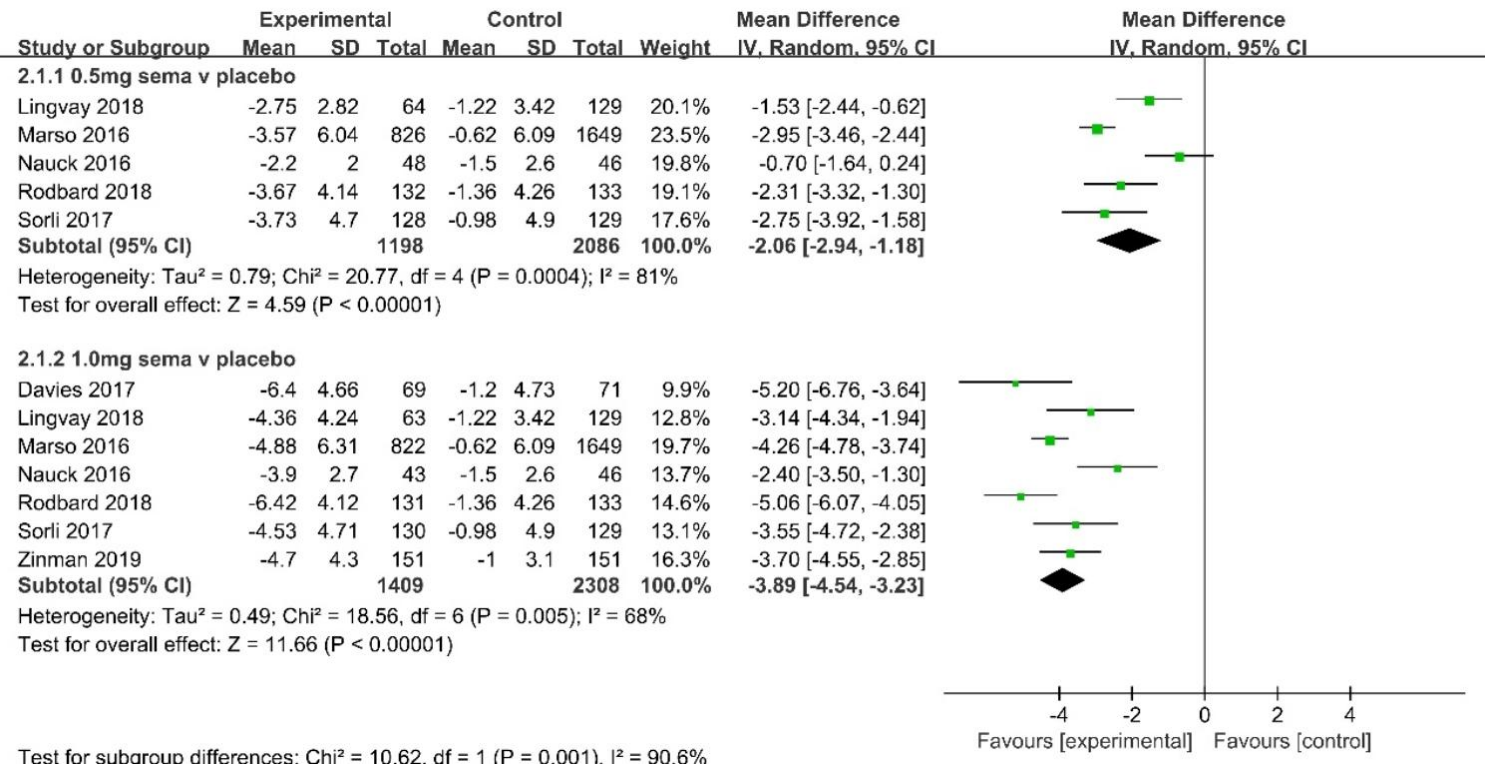
Mean difference of change in body weight (kg) between Semaglutide and placebo

Analyses for the change in body weight indicated a statistically significant reduction favoring semaglutide compared to placebo [MD -2.32 kg, 95% CI (-2.67, -1.96); *I*^*2*^ = 81%, 5 studies; MD -3.98 kg, 95% CI (-4.32, -3.64); *I*^*2*^ = 68%, 7 studies for semaglutide 0.5 and 1 mg, respectively] (Fig. 4).

**Fig. 5:**
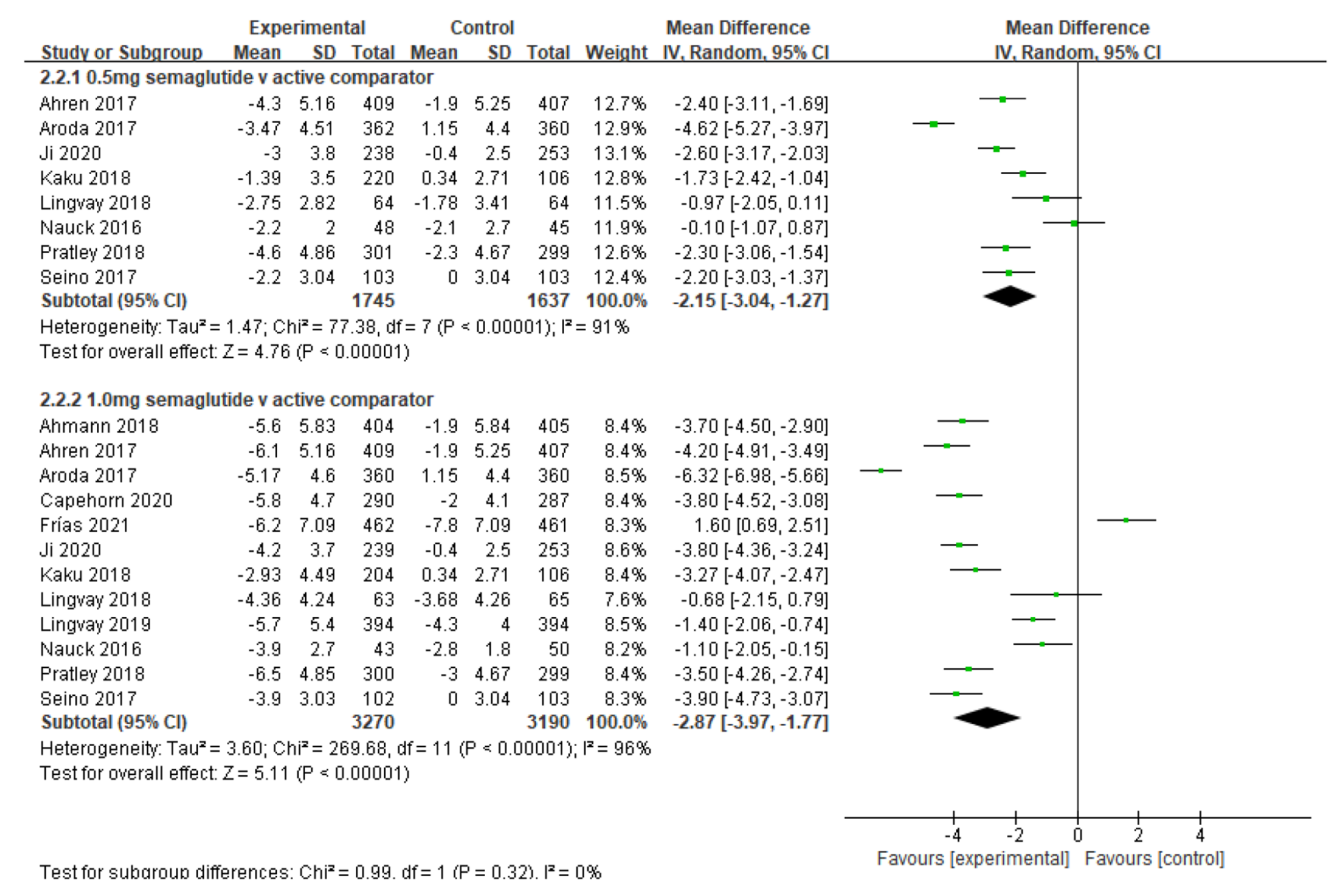
Mean difference of change in body weight (kg) between semaglutide and active comparator

Similarly, compared to other antidiabetic agents, semaglutide 0.5 and 1.0 mg lowered body weight by 2.15 kg [95% CI (-3.04, -1.27), *I*^*2*^ = 91%, 8 studies], and by 2.87 kg [95% CI (-3.97, -1.77), *I*^*2*^ = 94%, 12 studies], respectively (Fig. 5).

In the subgroup analysis, compared with GLP-1 RAs, body weight significantly decreased with 1.0 mg semaglutide, whereas no significant difference was detected between semaglutide 0.5 mg and GLP-1 RAs. At the same time, compared with sitagliptin, the reduction in body weight was notably greater in both doses of semaglutide. Of note, compared with 1.0 mg semaglutide, tirzepatide was associated with a significantly stronger reduction in body weight (Table S3).

#### Fasting plasma glucose

Reduction in FPG levels followed the same trend as that of HbA1c, semaglutide 0.5 mg reduced FPG by 1.34 mmol/L [95% CI (-1.85, -0.83), *I*^*2*^ = 76%, 5 studies]; this effect was enhanced with semaglutide 1 mg [MD -2.00 mmol/L, 95% CI (-2.52, -1.48), *I*^*2*^ = 85%, 7 studies] compared to placebo (Fig. S3).

Compared with the active comparator, treatment with 0.5 mg and 1.0 mg semaglutide reduced FPG by 0.83 mmol/L [95% CI (-1.29, -0.36), *I*^*2*^ = 91%, 8 studies], and by 0.92 mmol/L [95% CI (-1.43, -0.42), *I*^*2*^ = 96%, 12 studies] (Fig. S4).

Subgroup analyses revealed that semaglutide lowered FPG significantly compared with GLP-1 RAs and sitagliptin. It’s worth noting that compared to semaglutide, tirzepatide led to significant FPG reductions (Table S3).

#### Blood pressure

Compared with placebo or active comparators, treatment with semaglutide was associated with a reduction in SBP (Figs. S5 and S6).

The results of subgroup analysis showed that, compared with other GLP-1 RAs, semaglutide 1 mg appeared to have significantly reduced blood pressure compared to the other GLP-1 RAs. In addition, semaglutide was related to a significantly stronger reduction in SBP than sitagliptin (Table S3).

In our analysis of changes in diastolic blood pressure, there was no difference between semaglutide and the control group (Figs S7, S8).

#### Self-measured plasma glucose

Against placebo and the active comparator, semaglutide showed greater SMPG reduction. (Figs S9, S10).

In the subgroup analysis, when compared with GLP-1 RAs, the change in SMPG was notably greater in the semaglutide group. At the same time, two trials [15, 22] comparing semaglutide to sitagliptin reported a reduction in SMPG. Pooled results showed a statistically significant decrease in SMPG favoring semaglutide compared with sitagliptin (Table S3).

#### Proportion of patients achieving glycaemic targets

A greater percentage of patients achieved HbA1c<7%, HbA1c ≤ 6.5%, HbA1c < 7.0% without hypoglycaemia or weight gain with semaglutide 0.5 and 1.0 mg than with placebo or the active comparator (Figs S11-S16).

Comparing semaglutide 0.5 mg and GLP-1 RAs, the RR (95% CI) were 1.13 (0.90, 1.43) and 0.91 (0.32,2.55) in the number of patients achieving HbA1c < 7.0% and ≤ 6.5% respectively (Table S3).

### Tolerability outcomes

#### Adverse events

In comparison with the placebo, treatment with semaglutide did not increase the incidence of any adverse events, serious adverse events (Figs S21-S24). Results were similar for semaglutide 1 mg when compared to placebo. However, results for semaglutide 0.5 mg showed an increase in the incidence of serious adverse events compared with the active comparator. There was no significant difference found between semaglutide and GLP-1 RAs or sitagliptin (Table S3).

#### Severe or blood glucose-confirmed hypoglycaemia

There was no statistically significant difference in the incidence of severe or blood glucose-confirmed hypoglycaemia between semaglutide and placebo.

Similarly, the incidence of hypoglycaemia was not significantly different between semaglutide 1.0 mg and the active comparator. However, semaglutide 0.5 mg was associated with a lower incidence of hypoglycemia than the active comparator [RR 0.58, 95% CI (0.37,0.89); *I*^*2*^ = 0%, 7 studies] (Figs S25-S26).

Subgroup analyses showed no significant difference in the occurrence of hypoglycemia between semaglutide and GLP-1 RAs or sitagliptin (Table S3).

#### Gastrointestinal adverse events

Gastrointestinal symptoms included nausea, diarrhea, and vomiting. Semaglutide increased the risk of nausea, vomiting, and diarrhea significantly compared with placebo or other antidiabetic drugs. Semaglutide was associated with a slightly increase in nausea compared with GLP-1 RAs [RR 1.76, 95% CI (1.22, 2.54); *I*^*2*^ = 0%, 3 studies; RR 1.65, 95% CI (1.04, 2.62); *I*^*2*^ = 72%, 5 studies for semaglutide 0.5 and 1 mg, respectively] and sitagliptin [RR 3.74, 95% CI (1.80, 7.76); *I*^*2*^ = 39%, 3 studies; RR 5.92, 95% CI (1.78, 19.75); *I*^*2*^ = 74%, 3 studies for semaglutide 0.5 and 1 mg, respectively], except for the incidence of diarrhea and vomiting, which did not differ between semaglutide 1.0 mg and GLP-1 RAs (Table S3).

#### Acute Pancreatitis

In this meta-analysis, no difference was found between the semaglutide and control groups for the incidence of acute pancreatitis (AP). For subgroup analysis, semaglutide had a lower incidence of acute pancreatitis compared with GLP-1 RAs, whereas sitagliptin had a higher incidence. But the comparison of the results of the subgroup analysis was not statistically significant (Table S3).

#### Diabetic retinopathy

Regardless of the type of control arms (placebo, active comparator, GLP-1 RAs, and sitagliptin) or the treatment dose, semaglutide was not associated with an increase in the incidence of diaabetic retinopathy (DR) (Table S3).

## Discussion

This updated meta-analysis, including 17 RCTs that identified a further 5 RCTs, was conducted to comprehensively evaluate the efficacy and tolerability of subcutaneous semaglutide compared with placebo or other antidiabetic medications. In general, the results of this meta-analysis suggested that semaglutide showed a superior ability for glycemic lowering, body weight reduction, and blood pressure control compared with placebo or other hypoglycemic agents. Similar results were observed in the subgroup analysis comparing sitagliptin and GLP-1 RAs, except for body weight and SBP, which did not differ between the semaglutide 0.5 mg and GLP-1 RAs. Regarding tolerability outcomes, semaglutide did not increase the incidence of AEs, SAEs, or severe or BG-confirmed hypoglycaemia compared to placebo, which is consistent with the subgroup analysis. While semaglutide 0.5 mg can slightly decrease the risk of hypoglycaemia compared to an active comparator. However, treatment with semaglutide was associated with an increased risk of nausea, diarrhea and vomiting, but did not increase the risk of acute pancreatitis and diabetic retinopathy.

The long-term goal of diabetes management is to prevent chronic complications, improve the quality of life, and prolong life through great metabolic control [33]. Meanwhile, the United Kingdom Prospective Diabetes Study demonstrated that reaching and maintaining the blood glucose plays an important role in reducing the diabetic complications [34, 35]. To our knowledge, our findings were consistent with conclusion of previous meta-analyses [8–10] which were expressed the similarly improvement effects of the semaglutide on HbA1c. Moreover, SUSTAIN CHINA trial [15] found that semaglutdie made a more preferable effect on glycemic control than sitagliplin in Chinese. However, SURPASS-2 trial learned that tirzepatide appears to be superior to semaglutide in HbA1c and body weight control. The success of the challenge to semaglutide means that a new revolution is about to start, and tirzepatide, as the leader of the dual-target agonist class of hypoglycemic agents, is about to become the core competitor in the subsequent hypoglycemic field [16].

Data derived from the Framingham Heart Study suggested that T2D patients have a 2.5-fold increase in the cardiovascular disease (CVD) [36]. According to the latest statistics from the American Heart Association guideline, main CVD risk factors included high level of blood glucose, body weight, and SBP [37]. Lifestyle changes, such as exercise, can reduce the CVD risk. Exercise not only increases the number and sensitivity of insulin receptors on cell membranes but also improves insulin resistance, which can control blood glucose, indirectly [38–40]. From this meta-analysis, semaglutide expressed better effects on weight loss compared liraglutide and placebo [41]. The potential mechanism is that semaglutide delays gastric motility and activates gastric mechanoreceptors, which in turn inhibit the satiety center in the brainstem [42]. Furthermore, the STEP trials [43–46] validated that smeglutide had a significant weight loss effect. Therefore, subcutaneous semaglutide (Wegovy) has been approved by the FDA as an adjunct to a low-calorie diet and enhanced exercise for chronic weight management in obese or overweight adults.

In addition, previous results reported that semaglutide can significantly reduce SBP level compared with counterparts in the SUSTAIN series of clinical trials, except for SUSTAIN 8 trial [47]. In summary, semaglutide significantly reduces blood glucose, body weight, and SBP, thereby exerting an indirect cardiovascular protective effect. Furthermore, there is accumulating evidence that semaglutide reduces the expression of inflammatory factors in atherosclerotic mice, which directly protecting the cardiovascular system [48].

Gastrointestinal disorders were the most reported treatment-related AEs with semaglutide, which is consistent with other GLP-1 RAs [49]. In general, gastrointestinal events were dose-dependent, most of that were mild to moderate. Several possible mechanisms may explain the gastrointestinal adverse effects of GLP-1 RAs. First, GLP-1 RAs can bind to GLP-1 receptors in the gastrointestinal tract, which slowing gastric emptying [50]. Furthermore, GLP-1 RAs could aggravate anorexia and satiety through activating central GLP-1 receptors, thereby resulting in gastrointestinal events [51]. Therefore, further reduction of gastrointestinal discomfort can maximize the benefits of patients treated with GLP-1 RAs.

In this research, there were no statistical differences between semaglutide and other antidiabetic drugs in the incidence of AP and DR. Since the first case of pancreatitis in patients treated with exenatide in 2006, the tolerability of the pancreas for GLP-1 RAs has been a highly controversial topic in the past decade [52–54]. However, in agreement with our findings, there are several studies reporting that no association between treatment with GLP-1RAs and AP or DR [55–57]. In addition, some reports shown that semaglutide may play an important role in promoting cognitive function and neurodegenerative pathology [58, 59].

There are some limitations. Firstly, there was significant heterogeneity between the included trials that was not eliminated with sensitivity and subgroup analysis. These heterogeneities in findings could be attributed to the differences in the baseline characteristics of included trials, including race, treatment duration, background medication, control arms, and the actual dosage of semaglutide. Different antidiabetic agents used as controls could be the main reason for heterogeneity. It is worth mentioning that the dosage of semaglutide extracted from two dose-finding trials was less than the FDA-approved dose, which could underestimate the effectiveness of semaglutide and increase the heterogeneity [12, 28]. Secondly, two trials [25, 31] merely recruited patients from Japan, which caused potential heterogeneity. In addition, all included studies were funded by Novo Nordisk, and commercial sponsorship may increase bias risk. Finally, publication bias cannot be ignored when only published data were included. Against these shortcomings, it could be addressed by individual patient data meta-analysis of the efficacy and tolerability of semaglutide. Meanwhile, large, multi-center clinical trials in real medical world should be conducted to obtain stronger levels of evidence to better guide clinical treatment decisions in the future. Most importantly, although semaglutide has been validated as an important part of hypoglycemic regimen, treatment of T2D patient should be individualized based on demographic characteristics and personal circumstances.

## Conclusion

In conclusion, subcutaneous semaglutide appears to exhibite beneficial effects regarding the reduction of HbA1c, weight loss, and SBP. Treatment with subcutaneous semaglutide did not increase the risk of hypoglycemia but was associated with increased incidence of nausea, vomiting, and diarrhea.

### Electronic supplementary material

Below is the link to the electronic supplementary material.

Supplementary Material 1

## Data Availability

The data used to support the findings of this study are included in the article.
