# Supplementary material for: Efficacy and tolerability of the Subcutaneous Semaglutide for type 2 Diabetes patients: an updated systematic review and meta-analysis
Source: Diabetol Metab Syndr. 2023 Oct 28;15:218. doi: 10.1186/s13098-023-01195-7 (PMC10612199; doi:10.1186/s13098-023-01195-7)
Supplement: Supplementary file 1 — Supplementary Material 1 [file 13098_2023_1195_MOESM1_ESM.docx]

**Table S1.** Search strategy

| **#1** | **semaglutide [Supplementary Concept]** |
| --- | --- |
| **#2** | **semaglutide [Title/Abstract]** |
| **#3** | **NN9935 [Title/Abstract]** |
| **#4** | **#1 OR #2 OR #3** |
| **#5** | **Diabetes Mellitus, Type II [Mesh]** |
| **#6** | **Diabetes Mellitus, Noninsulin-Dependent [Mesh]** |
| **#7** | **T2D [Title/Abstract]** |
| **#8** | **#5 OR #6 OR #7** |
| **#9** | **#4 AND #8** |

**Table S2.** Selection Criteria

| **Inclusion criteria：** |
| --- |
| (1) RCTs that compared subcutaneous semaglutide with placebo or any other active comparator in adults with T2D; |
| (2) the diagnosis of T2DM is consistent with the definition put forward by WHO in 1999; |
| (3) treatment duration≥12 weeks; |
| (4) at least one of the following outcomes was reported in a trial: change in HbA1c, change in body weight, change in FPG, change in SMPG, number of particioants achieving HbA1c＜7.0%, AEs, SAEs and hypoglycaemic events; |
| (5) the research results were published in English or Chinese. |
| **exclusion criteria：** |
| (1) studies that did not aimed at the effectiveness and safety of semaglutide; |
| (2) RCTs not for T2DM, but obesity, impaired glucose tolerance (IGT), gestational dibetes or type1 diabetes; |
| (3) trials conducted with animals or cells; |
| (4) trials that incomplete data； |
| (5) published in the form of abstracts, short communications, or brief reports,systematic review and meta-analysis, hoc-analysis, pharmacoeconomics research or letter and comments. |
| (6) **duplicate studies.** |

Records and studies included in systematic review and meta-analysis (n=17)

1063 Records identified through searching

PubMed (n=300)

Science Direct (n=63)

Cochrane Library (n=368)

Clinicaltrial (n=30)

Springer (n=77)

OVID (n=158)

CNKICNKI (n=36)

WangFang Data (n=17)

VIP (n=14)

Duplicates (n=238)

Records screened at title and abstract (n=825)

Records excluded at title and abstract (n=785)

Full-text screened (n=40)

23 Full-text records excluded :

Clinicaltrials.gov record with no results (n=5)

Unable to get full text (n=7)

Results not fully published (n=1)

Oral Semaglutide (n=10)

**Fig S1** Flowchart of literature search


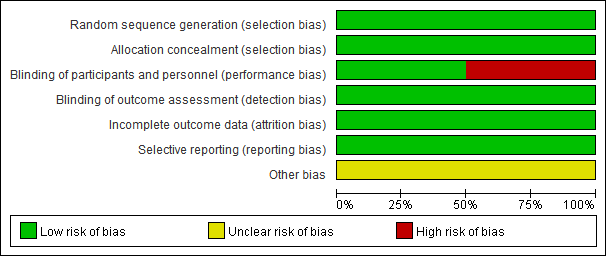


**FIG.S2** Risk of bias graph: review authors' judgements about each risk of bias item presented as percentages across all included studies.


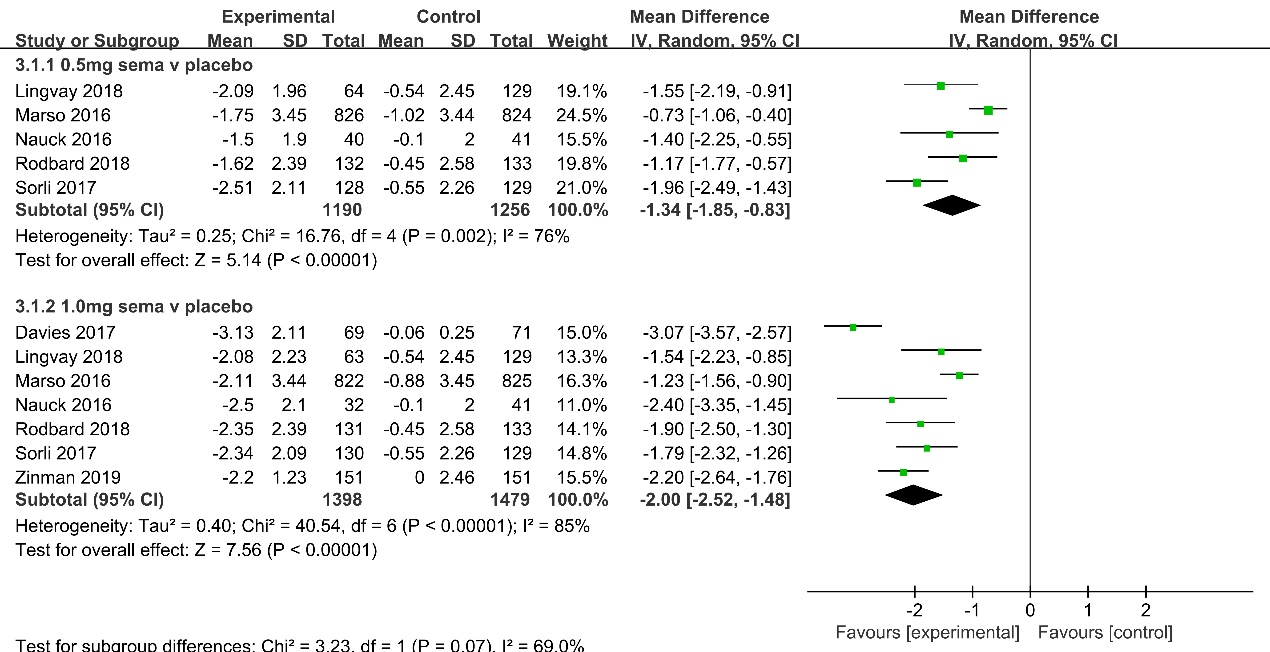


**FIG.S3** Mean difference of change in FPG (mmol/L) between Semaglutide and placebo.


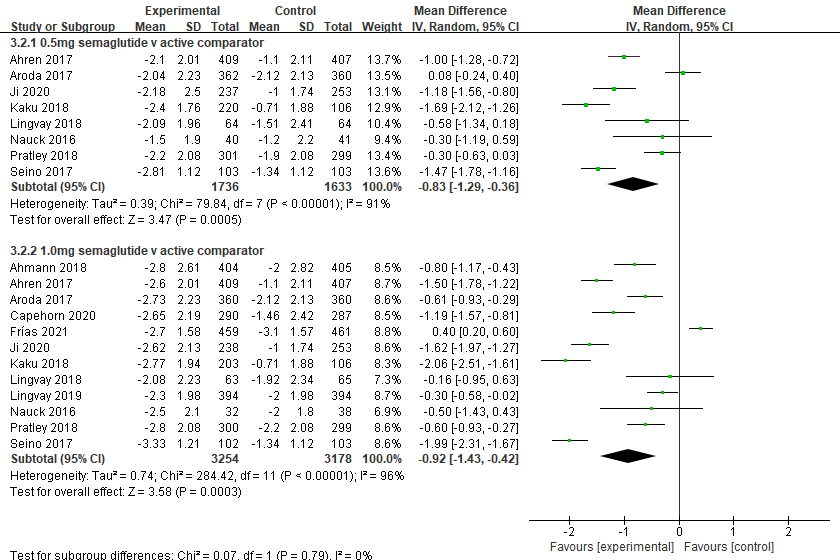


**FIG.S4** Mean difference of change in FPG (mmol/L) between Semaglutide and active comparator.


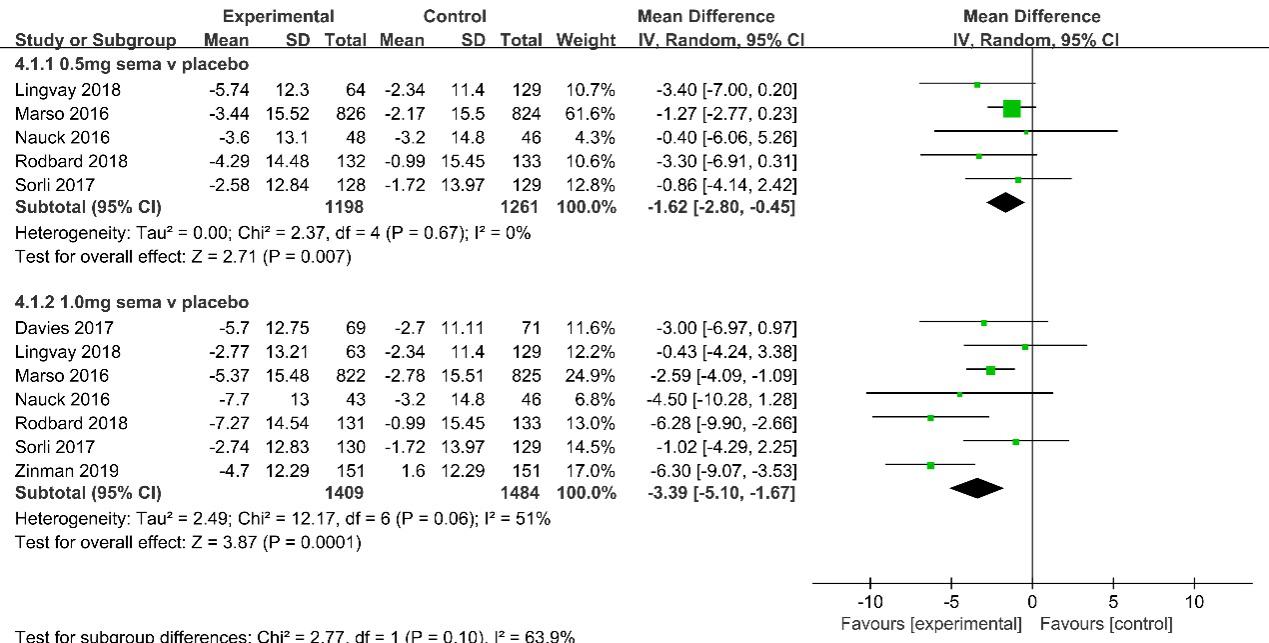


**FIG.S5** Mean difference in change in SBP (mm Hg) between Semaglutide and placebo.


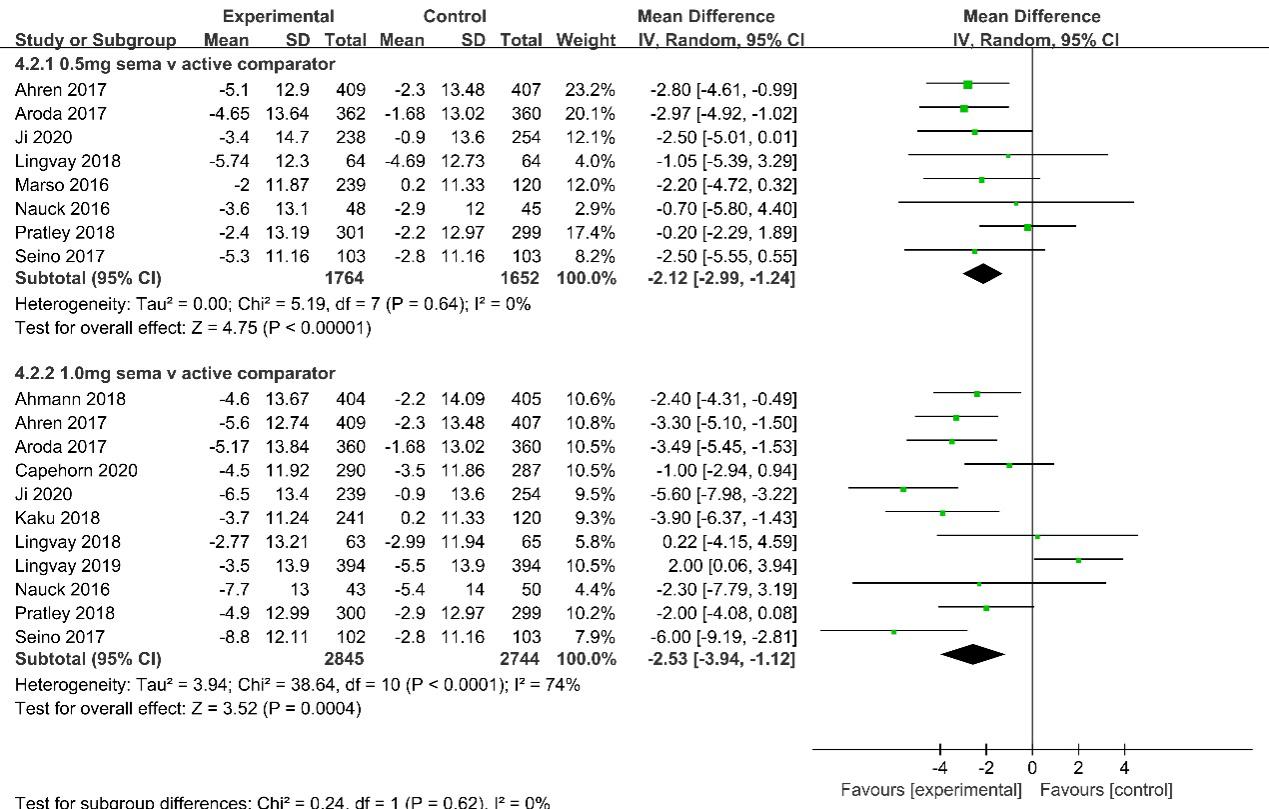


**FIG.S6** Mean difference in change in SBP (mm Hg) between Semaglutide and active comparator.


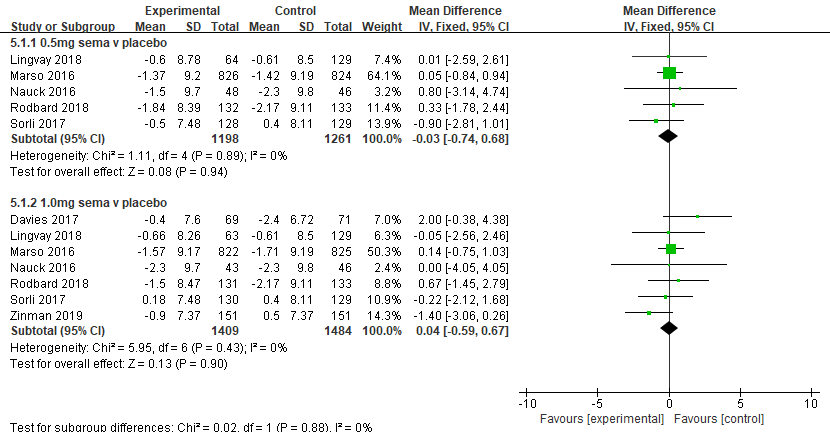
**FIG.S7** Mean difference in change in DBP (mm Hg) between Semaglutide and placebo.


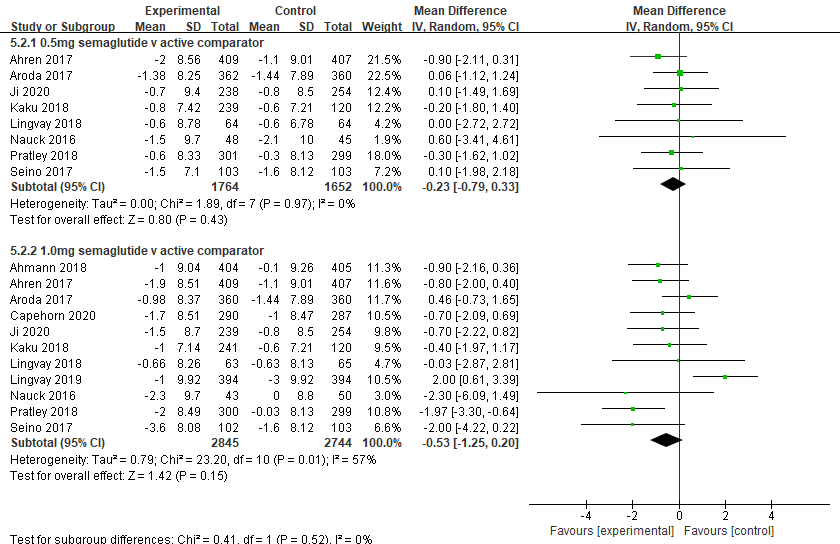
**FIG.S8** Mean difference in change in DBP (mm Hg) between Semaglutide and active comparator.


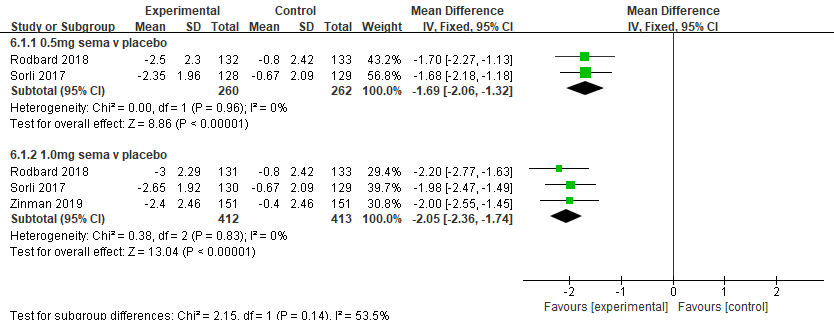
**FIG.S9** Mean difference in change in SMPG (mmol/L) between Semaglutide and placebo.


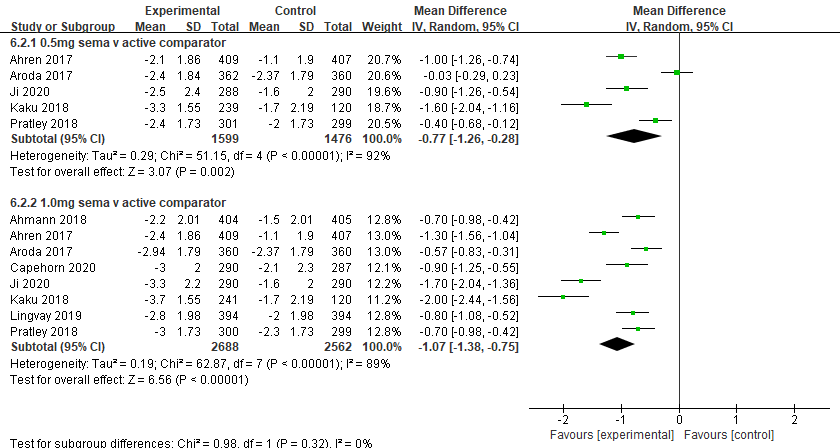
**FIG.S10** Mean difference in change in SMPG (mmol/L) between Semaglutide and active comparator.


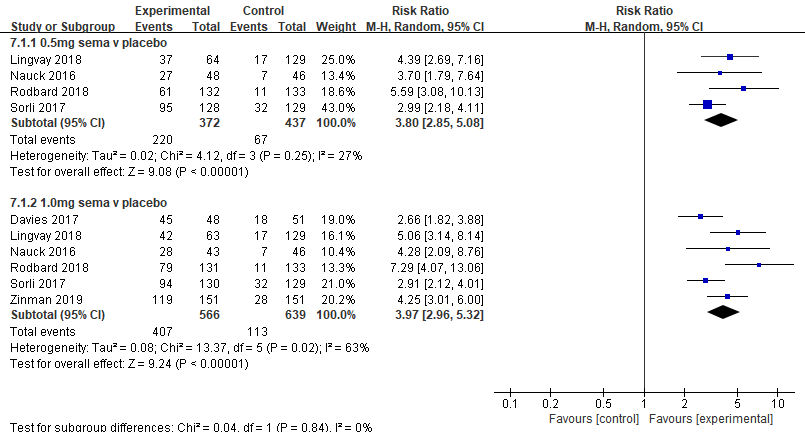
**FIG S11** Number of patients achieving haemoglobin A1c< 7.0% between semaglutide and placebo.


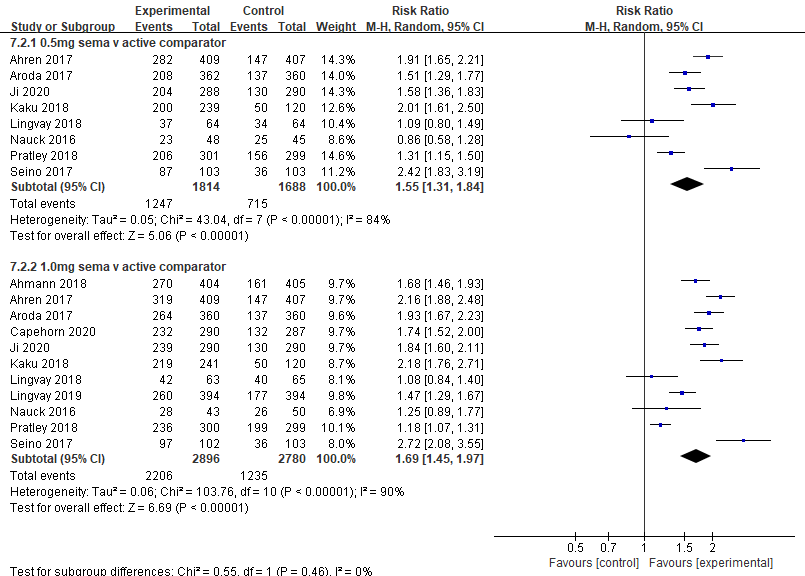
**FIG S12** Number of patients achieving haemoglobin A1c< 7.0% between semaglutide and active comparator.


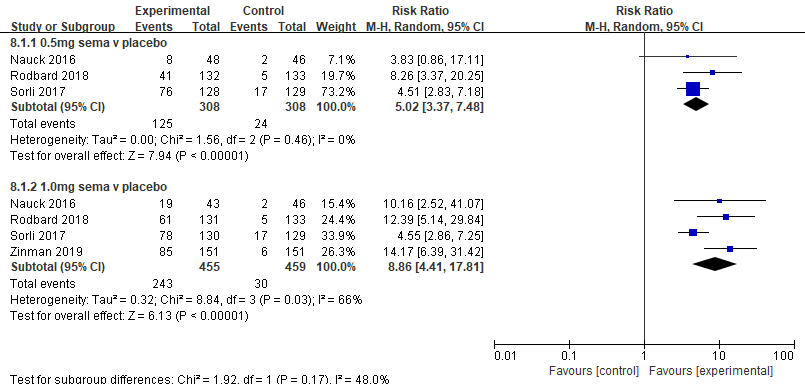
**FIG S13** Number of patients achieving haemoglobin A1c<6.5% between semaglutide and placebo.


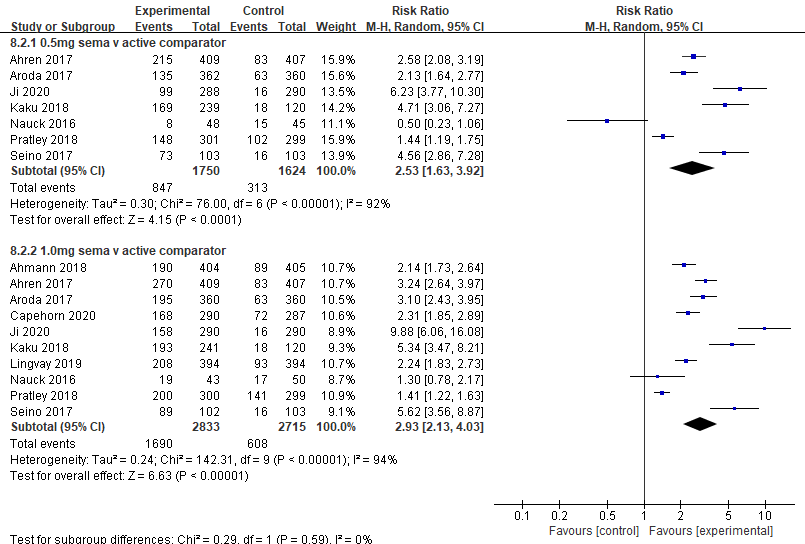
**FIG S14** Number of patients achieving haemoglobin A1c<6.5% between semaglutide and active comparator.


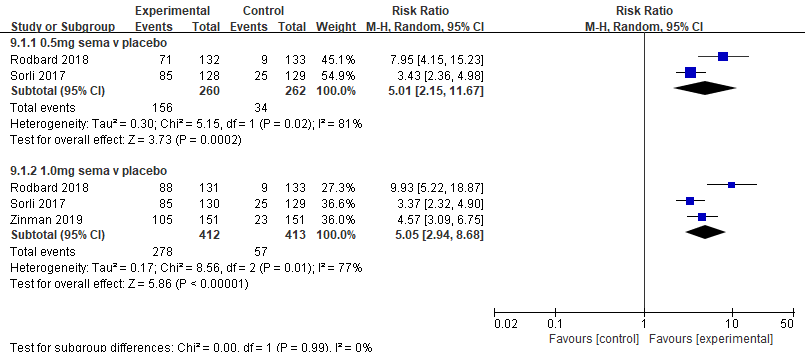


**FIG S15** Number of patients achieving haemoglobin A1c<7.0% without hypoglycaemia or weight gain between semaglutide and placebo.


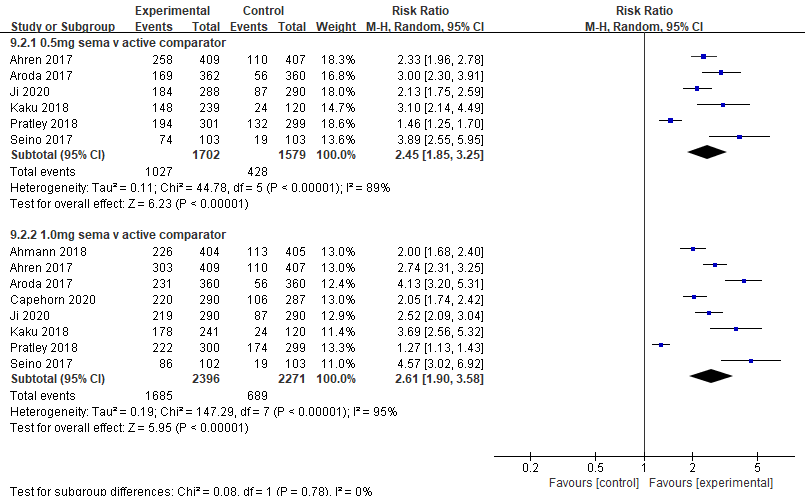


**FIG S16** Number of patients achieving haemoglobin A1c<7.0% without hypoglycaemia or weight gain between semaglutide and active comparator.
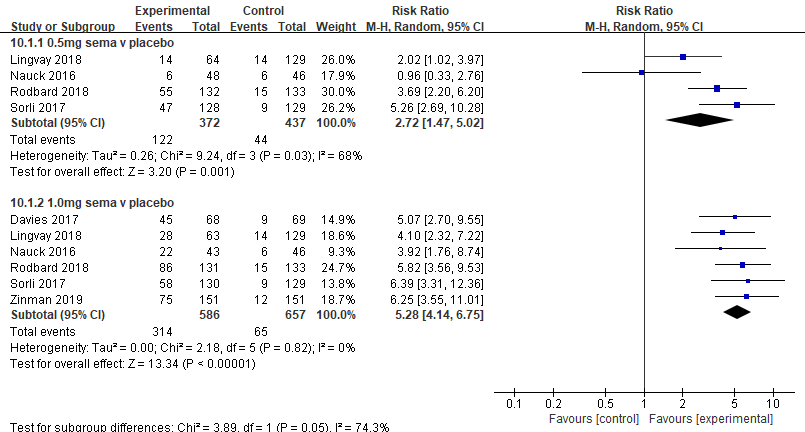
**FIG S17** Number of patients achieving body weight loss≥5% between semaglutide and placebo.


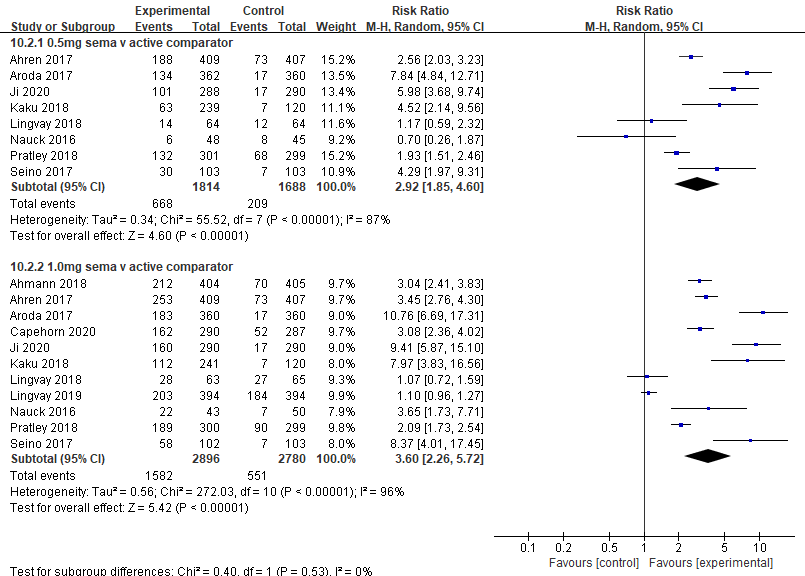
**FIG S18** Number of patients achieving body weight loss≥5% between semaglutide and active comparator.


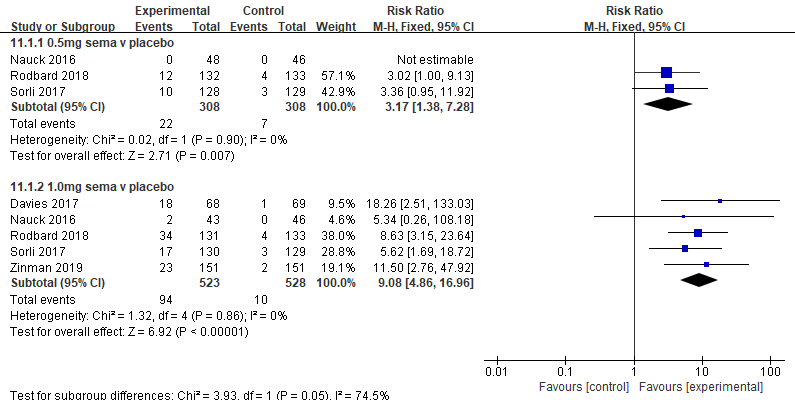
**FIG S19** Number of patients achieving body weight loss≥10% between semaglutide and placebo.


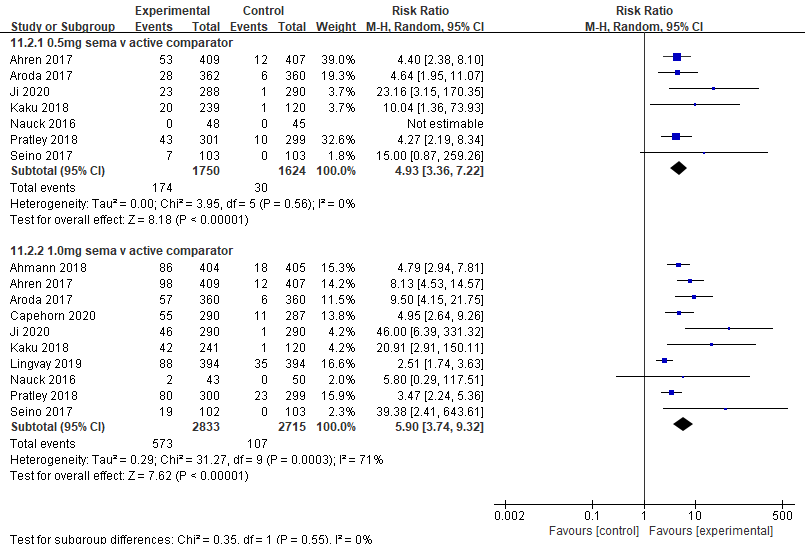
**FIG S20** Number of patients achieving body weight loss≥10% between semaglutide and active comparator.
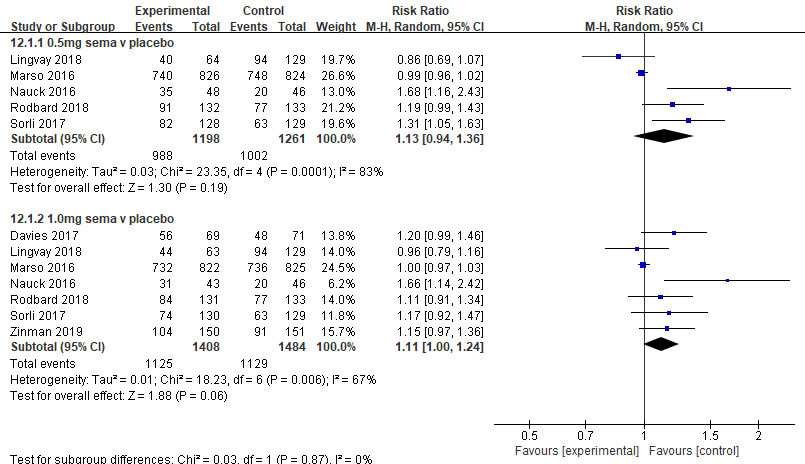
**Figure S21** Risk Ratio (RR) for incidence of any adverse events between semaglutide and placebo.


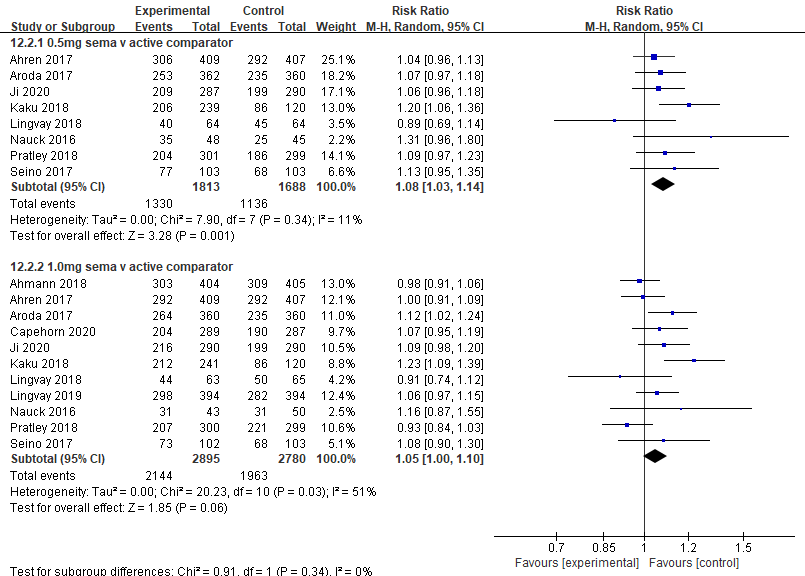


**Figure S22** Risk Ratio (RR) for incidence of any adverse events between semaglutide and active comparator.
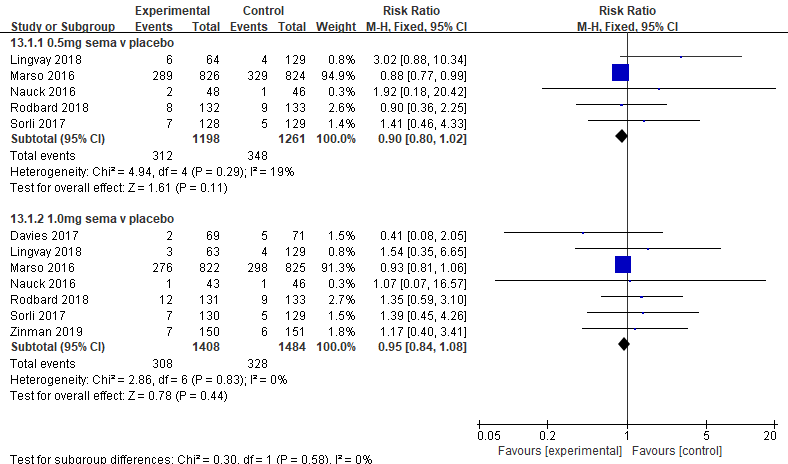
**Figure S23** Risk Ratio (RR) for incidence of serious adverse events between semaglutide and placebo.


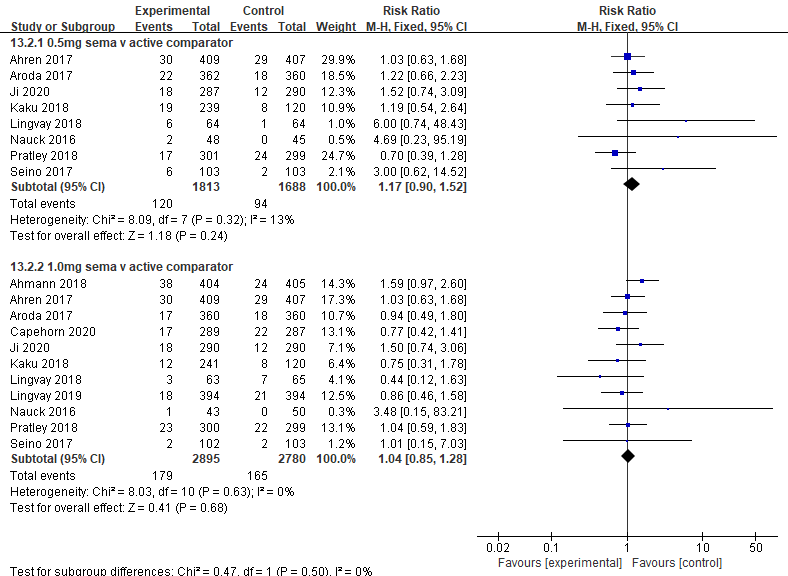


**Figure S24** Risk Ratio (RR) for incidence of serious adverse events between semaglutide and active comparator.


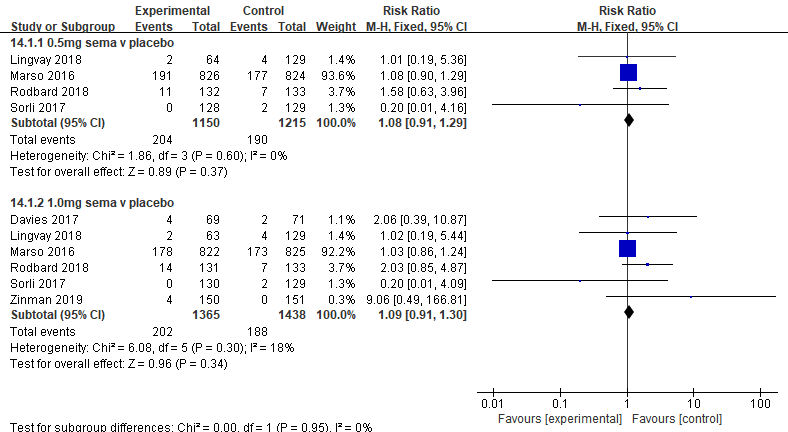


**Figure S25** Risk Ratio (RR) for incidence of Severe or blood glucose-confirmed hypoglycaemia events between semaglutide and placebo.


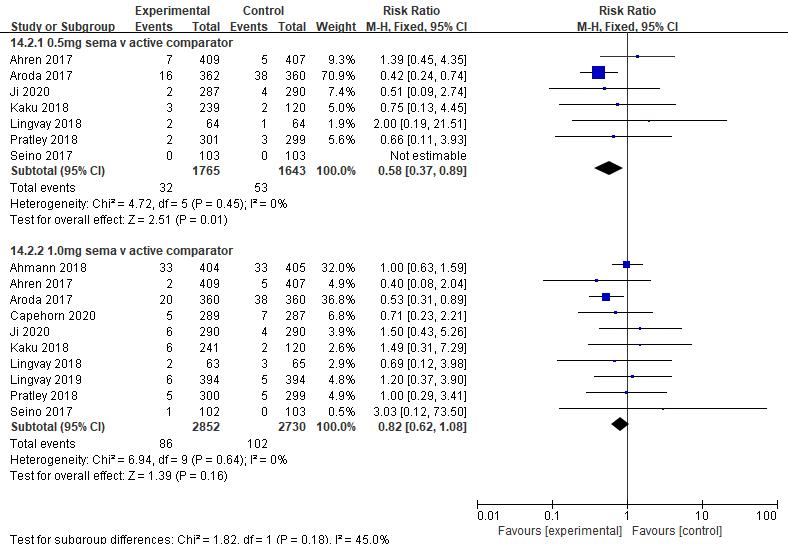


**Figure S26** Risk Ratio (RR) for incidence of Severe or blood glucose-confirmed hypoglycaemia events between semaglutide and active comparator.


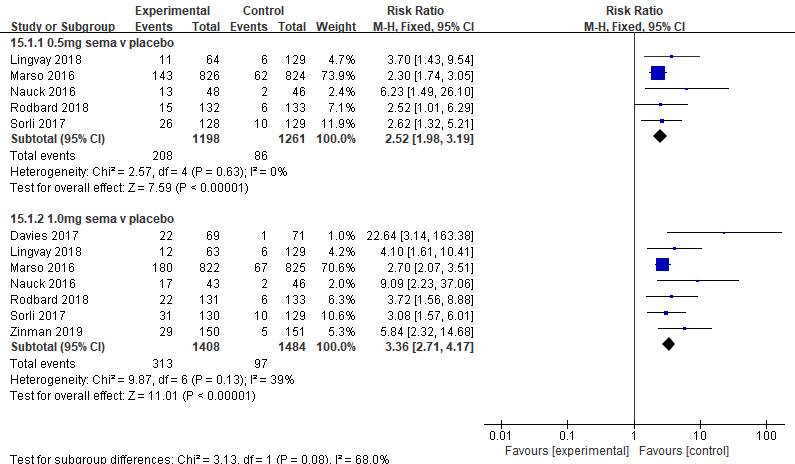


**Figure S27** Risk Ratio (RR) for incidence of nausea events between semaglutide and placebo.


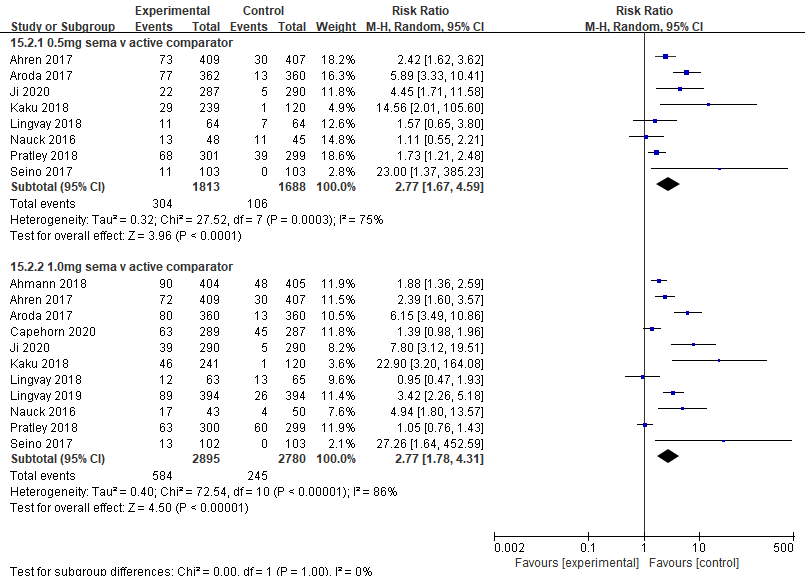


**Figure S28** Risk Ratio (RR) for incidence of nausea events between semaglutide and active comparator.


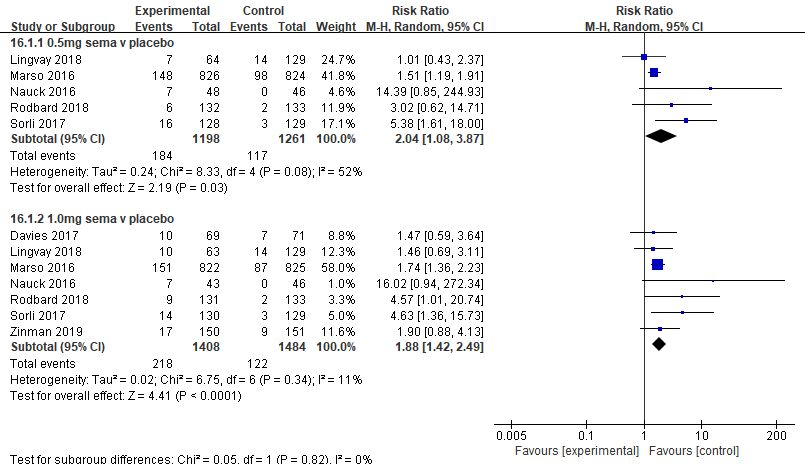


**Figure S29** Risk Ratio (RR) for incidence of diarrhea events between semaglutide and placebo.


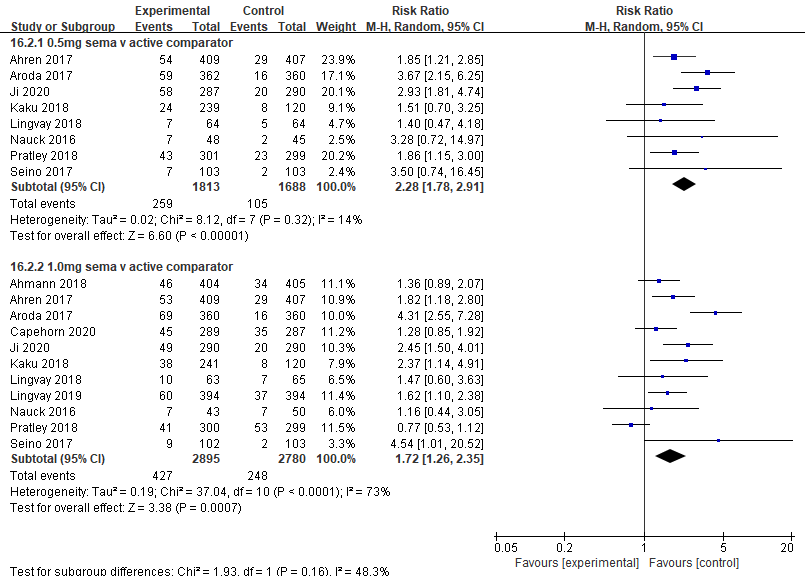


**Figure S30** Risk Ratio (RR) for incidence of diarrhea events between semaglutide and active comparator.


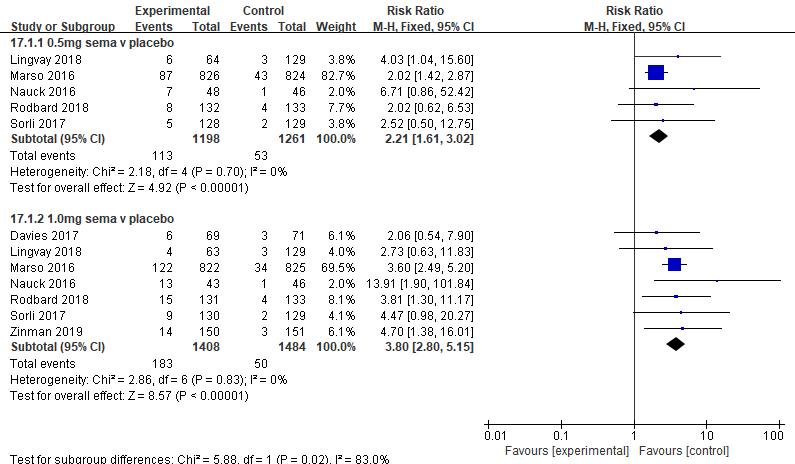


**Figure S31** Risk Ratio (RR) for incidence of vomiting events between semaglutide and placebo.


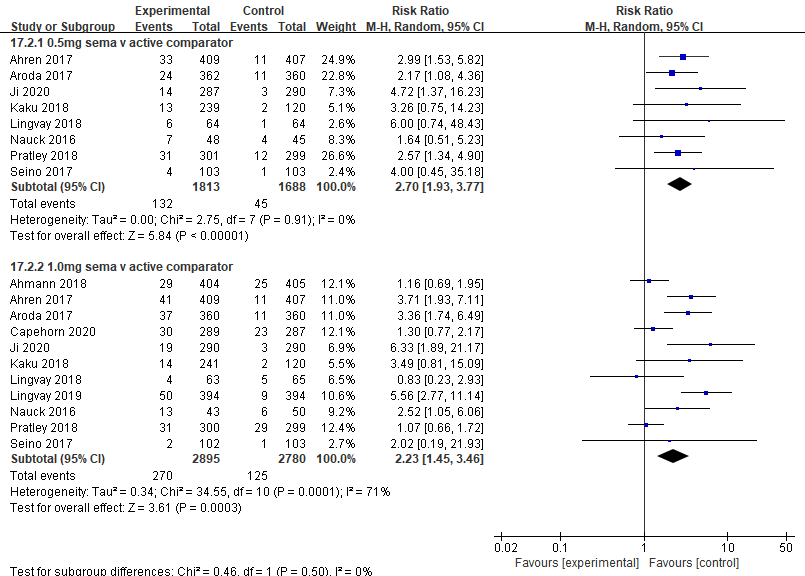


**Figure S32** Risk Ratio (RR) for incidence of vomiting events between semaglutide and active comparator.


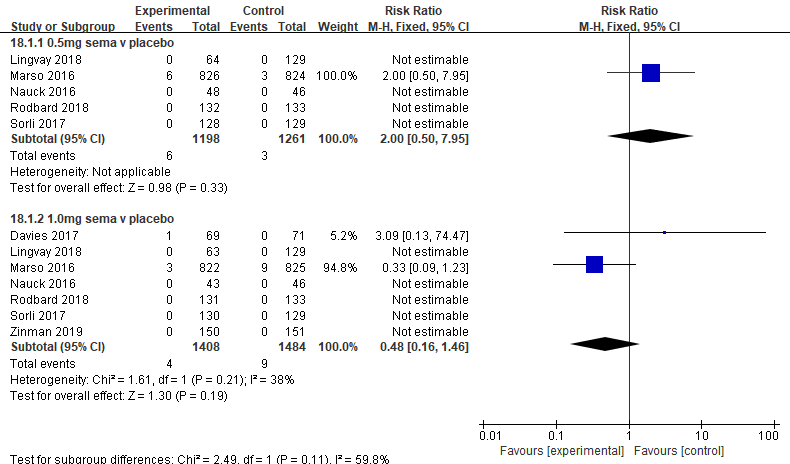


**Figure S33** Risk Ratio (RR) for incidence of acute pancreatitis events between semaglutide and placebo.


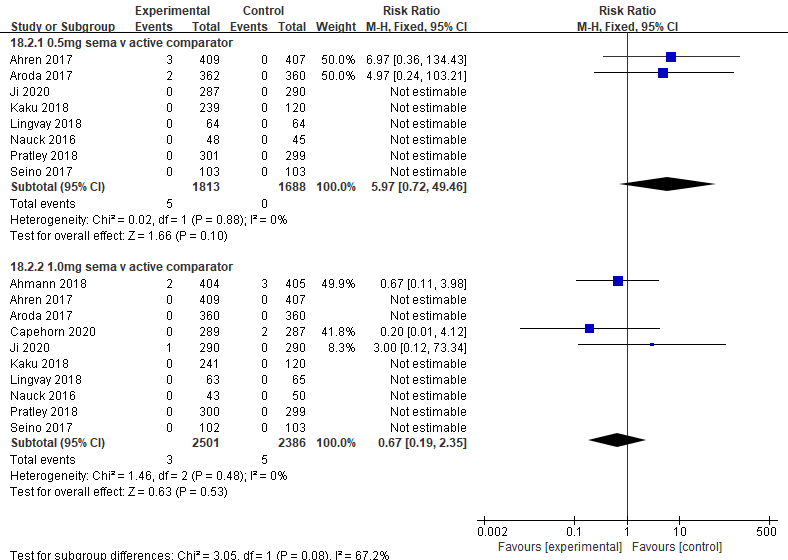


**Figure S34** Risk Ratio (RR) for incidence of acute pancreatitis events between semaglutide and active comparator.


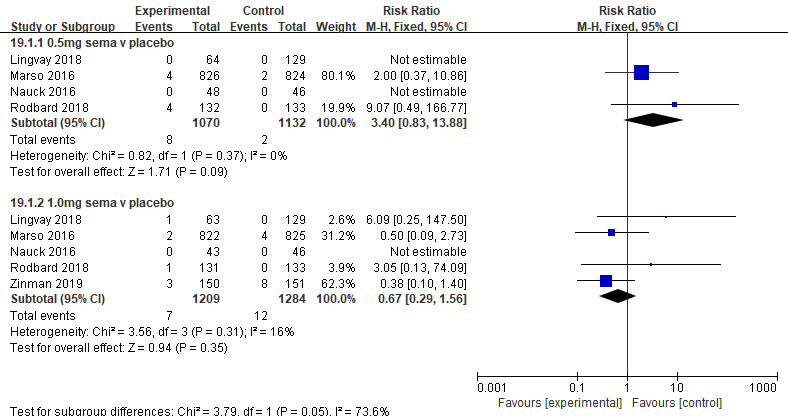


**Figure S35** Risk Ratio (RR) for incidence of diabetic retinopathy events between semaglutide and placebo.


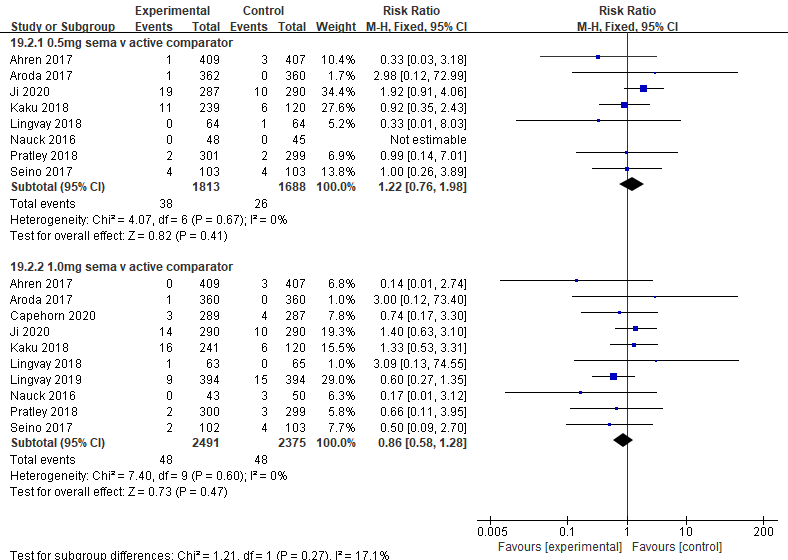


**Figure S36** Risk Ratio (RR) for incidence of diabetic retinopathy events between semaglutide and active comparator.

| **Table S3.** Summary of meta-analysis for efficacy and safety outcomes | | | | | | |
| --- | --- | --- | --- | --- | --- | --- |
| outcome | subgroup | Studies  included,  n | participants analysed | | MD/RR(95%CI) | I²,% |
|  |  |  | semaglutide | comparator |  |  |
| Reduction in HbA1c (%) | 0.5 mg semaglutide vs placebo | 5 | 1198 | 1261 | -0.97 (-1.33,-0.62) | 91 |
|  | 1.0 mg semaglutide vs placebo | 7 | 1409 | 1484 | -1.36 (-1.59,-1.13) | 84 |
|  | 0.5 mg semaglutide vs active comparator | 8 | 1742 | 1634 | -0.56 (-0.80,-0.32) | 91 |
|  | 1.0 mg semaglutide vs active comparator | 11 | 2706 | 2645 | -0.71 (-0.93,-0.50) | 93 |
|  | 0.5 mg semaglutide vs GLP-1 RAs | 3 | 413 | 408 | -0.20 (-0.46,-0.07) | 71 |
|  | 1.0 mg semaglutide vs GLP-1 RAs | 5 | 1100 | 1106 | -0.38 (-0.58,-0.18) | 79 |
|  | 0.5 mg semaglutide vs DPP-4i | 3 | 747 | 760 | -0.75 (-0.86,-0.65) | 89 |
|  | 1.0 mg semaglutide vs DPP-4i | 3 | 749 | 760 | -1.03 (-1.13,-0.93) | 90 |
|  | 1.0 mg semaglutide vs SGLT-2i | 1(canagliflozin) | 293 | 293 | -0.50 (-0.69,-0.31) | NA |
|  | 1.0 mg semaglutide vs insulinotropic polypeptide–GLP-1 RA | 1(Tirzepatide) | 461 | 461 | 0.23 (0.10, 0.36) | NA |
|  | 0.5 mg semaglutide vs Insulin | 1(Insulin glargine) | 362 | 360 | -0.38 (-0.53,-0.23) | NA |
|  | 1.0 mg semaglutide vs Insulin | 1(Insulin glargine) | 360 | 360 | -0.81 (-0.95,-0.67) | NA |
|  | 0.5 mg semaglutide vs additional OADs | 1 | 220 | 106 | -1.07 (-1.30,-0.84) | NA |
|  | 1.0 mg semaglutide vs additional OADs | 1 | 204 | 106 | -1.36 (-1.59,-1.13) | NA |
| Reduction in body weight (kg) | 0.5 mg semaglutide vs placebo | 5 | 1198 | 2086 | -2.32 (-2.67,-1.96) | 81 |
|  | 1.0 mg semaglutide vs placebo | 7 | 1409 | 2308 | -3.98 (-4.32,-3.64) | 68 |
|  | 0.5 mg semaglutide vs active comparator | 8 | 1745 | 1637 | -2.15 (-3.04,-1.27) | 91 |
|  | 1.0 mg semaglutide vs active comparator | 11 | 2808 | 2729 | -3.29 (-4.19,-2.39) | 94 |
|  | 0.5 mg semaglutide vs GLP-1 RAs | 3 | 413 | 408 | -1.15 (-2.51,0.21) | 84 |
|  | 1.0 mg semaglutide vs GLP-1 RAs | 5 | 1100 | 1106 | -2.65 (-3.81,-1.49) | 88 |
|  | 0.5 mg semaglutide vs DPP-4i | 3 | 750 | 763 | -2.45 (-2.84,-2.06) | 0 |
|  | 1.0 mg semaglutide vs DPP-4i | 3 | 750 | 763 | -3.94 (-4.33,-3.55) | 0 |
|  | 1.0 mg semaglutide vs SGLT-2i | 1(canagliflozin) | 394 | 394 | -1.40 (-2.06,-0.74) | NA |
|  | 1.0 mg semaglutide vs insulinotropic polypeptide–GLP-1 RA | 1(Tirzepatide) | 462 | 461 | 1.60 (0.69, 2.51) | NA |
|  | 0.5 mg semaglutide vs Insulin | 1(Insulin glargine) | 362 | 360 | -4.62 (-5.27,-3.97) | NA |
|  | 1.0 mg semaglutide vs Insulin | 1(Insulin glargine) | 360 | 360 | -6.32 (-6.98,-5.66) | NA |
|  | 0.5 mg semaglutide vs additional OADs | 1 | 220 | 106 | -1.73 (-2.42,-1.04) | NA |
|  | 1.0 mg semaglutide vs additional OADs | 1 | 204 | 106 | -3.27 (-4.07,-2.47) | NA |
| Reduction in FPG  (mmol/L) | 0.5 mg semaglutide vs placebo | 5 | 1190 | 1256 | -1.34 (-1.85,-0.83) | 76 |
|  | 1.0 mg semaglutide vs placebo | 7 | 1398 | 1479 | -2.00 (-2.52,-1.48) | 85 |
|  | 0.5 mg semaglutide vs active comparator | 8 | 1736 | 1633 | -0.83 (-1.29,-0.36) | 91 |
|  | 1.0 mg semaglutide vs active comparator | 11 | 2795 | 2717 | -1.06 (-1.46,-0.67) | 92 |
|  | 0.5 mg semaglutide vs GLP-1 RAs | 3 | 405 | 404 | -0.34 (-0.63,-0.05) | 0 |
|  | 1.0 mg semaglutide vs GLP-1 RAs | 5 | 1089 | 1094 | -0.75 (-1.06,-0.43) | 52 |
|  | 0.5 mg semaglutide vs DPP-4i | 3 | 749 | 763 | -1.21 (-1.51,-0.92) | 59 |
|  | 1.0 mg semaglutide vs DPP-4i | 3 | 749 | 763 | -1.70 (-1.99,-1.40) | 62 |
|  | 1.0 mg semaglutide vs SGLT-2i | 1(canagliflozin) | 394 | 394 | -0.30 (-0.58,-0.02) | NA |
|  | 1.0 mg semaglutide vs insulinotropic polypeptide–GLP-1 RA | 1(Tirzepatide) | 459 | 461 | 0.40 (0.20, 0.60) | NA |
|  | 0.5 mg semaglutide vs Insulin | 1(Insulin glargine) | 362 | 360 | 0.08 (-0.24,0.40) | NA |
|  | 1.0 mg semaglutide vs Insulin | 1(Insulin glargine) | 360 | 360 | -0.61 (-0.93,-0.29) | NA |
|  | 0.5 mg semaglutide vs additional OADs | 1 | 220 | 106 | -1.69 (-2.12,-1.26) | NA |
|  | 1.0 mg semaglutide vs additional OADs | 1 | 203 | 106 | -2.06 (-2.51,-1.61) | NA |
| Change in SMPG  (mmol/L) | 0.5 mg semaglutide vs placebo | 2 | 260 | 262 | -1.69 (-2.06,-1.32) | 0 |
|  | 1.0 mg semaglutide vs placebo | 3 | 412 | 413 | -2.05 (-2.36,-1.74) | 0 |
|  | 0.5 mg semaglutide vs active comparator | 5 | 1599 | 1476 | -0.77 (-1.26,-0.28) | 92 |
|  | 1.0 mg semaglutide vs active comparator | 8 | 2688 | 2562 | -1.07 (-1.38,-0.75) | 89 |
|  | 0.5 mg semaglutide vs GLP-1 RAs | 1 | 301 | 299 | -0.40 (-0.68,-0.12) | NA |
|  | 1.0 mg semaglutide vs GLP-1 RAs | 3 | 994 | 991 | -0.75 (-0.92,-0.58) | 0 |
|  | 0.5 mg semaglutide vs DPP-4i | 2 | 697 | 697 | -0.97 (-1.18,-0.76) | 0 |
|  | 1.0 mg semaglutide vs DPP-4i | 2 | 699 | 697 | -1.48 (-1.87,-1.09) | 70 |
|  | 1.0 mg semaglutide vs SGLT-2i | 1(canagliflozin) | 394 | 394 | -0.80 (-1.08,-0.52) | NA |
|  | 0.5 mg semaglutide vs Insulin | 1(Insulin glargine) | 362 | 360 | -0.03 (-0.29,0.23) | NA |
|  | 1.0 mg semaglutide vs Insulin | 1(Insulin glargine) | 360 | 360 | -0.57 (-0.83,-0.31) | NA |
|  | 0.5 mg semaglutide vs additional OADs | 1 | 239 | 120 | -1.60 (-2.04,-1.16) | NA |
|  | 1.0 mg semaglutide vs additional OADs | 1 | 241 | 120 | -2.00 (-2.44,-1.56) | NA |
| Reduction in SBP  (mm Hg) | 0.5 mg semaglutide vs placebo | 5 | 1198 | 1261 | -1.62 (-2.80,-0.45) | 0 |
|  | 1.0 mg semaglutide vs placebo | 7 | 1409 | 1484 | -3.39 (-5.10,-1.67) | 51 |
|  | 0.5 mg semaglutide vs active comparator | 8 | 1764 | 1652 | -2.12 (-2.99,-1.24) | 0 |
|  | 1.0 mg semaglutide vs active comparator | 11 | 2845 | 2744 | -2.53 (-3.94,-1.12) | 74 |
|  | 0.5 mg semaglutide vs GLP-1 RAs | 3 | 413 | 408 | -0.40 (-2.17,1.37) | 0 |
|  | 1.0 mg semaglutide vs GLP-1 RAs | 5 | 1100 | 1106 | -1.69 (-2.77,-0.61) | 0 |
|  | 0.5 mg semaglutide vs DPP-4i | 3 | 750 | 764 | -2.66 (-3.98,-1.34) | 0 |
|  | 1.0 mg semaglutide vs DPP-4i | 3 | 750 | 764 | -4.45 (-5.76,-3.14) | 41 |
|  | 1.0 mg semaglutide vs SGLT-2i | 1(canagliflozin) | 394 | 394 | 2.00 (0.06,3.94) | NA |
|  | 0.5 mg semaglutide vs Insulin | 1(Insulin glargine) | 362 | 360 | -2.97 (-4.92,-1.02) | NA |
|  | 1.0 mg semaglutide vs Insulin | 1(Insulin glargine) | 360 | 360 | -3.49 (-5.45,-1.53) | NA |
|  | 0.5 mg semaglutide vs additional OADs | 1 | 239 | 120 | -2.20 (-4.72,0.32) | NA |
|  | 1.0 mg semaglutide vs additional OADs | 1 | 241 | 120 | -3.90 (-6.37,-1.43) | NA |
| Reduction in DBP  (mm Hg) | 0.5 mg semaglutide vs placebo | 5 | 1198 | 1261 | -0.03 (-0.74,0.68) | 0 |
|  | 1.0 mg semaglutide vs placebo | 7 | 1409 | 1484 | 0.04 (-0.59,0.67) | 0 |
|  | 0.5 mg semaglutide vs active comparator | 8 | 1764 | 1652 | -0.23 (-0.79,0.33) | 0 |
|  | 1.0 mg semaglutide vs active comparator | 11 | 2845 | 2744 | -0.53 (-1.25,0.20) | 57 |
|  | 0.5 mg semaglutide vs GLP-1 RAs | 3 | 413 | 408 | -0.18 (-1.31,0.96) | 0 |
|  | 1.0 mg semaglutide vs GLP-1 RAs | 5 | 1100 | 1106 | -1.16 (-1.88,-0.43) | 0 |
|  | 0.5 mg semaglutide vs DPP-4i | 3 | 750 | 764 | -0.42 (-1.29,0.45) | 0 |
|  | 1.0 mg semaglutide vs DPP-4i | 3 | 750 | 764 | -0.95 (-1.82,-0.08) | 0 |
|  | 1.0 mg semaglutide vs SGLT-2i | 1(canagliflozin) | 394 | 39 | 2.00 (0.61,3.39) | NA |
|  | 0.5 mg semaglutide vs Insulin | 1(Insulin glargine) | 362 | 360 | 0.06 (-1.12,1.24) | NA |
|  | 1.0 mg semaglutide vs Insulin | 1(Insulin glargine) | 360 | 360 | 0.46 (-0.73,1.65) | NA |
|  | 0.5 mg semaglutide vs additional OADs | 1 | 239 | 120 | -2.20 (-4.72,0.32) | NA |
|  | 1.0 mg semaglutide vs additional OADs | 1 | 241 | 120 | -3.90 (-6.37,-1.43) | NA |
| Number of participants achieving HbA1c <7.0% | 0.5 mg semaglutide vs placebo | 4 | 372 | 437 | 3.80 (2.85,5.08) | 27 |
|  | 1.0 mg semaglutide vs placebo | 6 | 566 | 639 | 3.97 (2.96,5.32) | 63 |
|  | 0.5 mg semaglutide vs active comparator | 8 | 1814 | 1688 | 1.55 (1.31,1.84) | 84 |
|  | 1.0 mg semaglutide vs active comparator | 11 | 2896 | 2780 | 1.69 (1.45,1.97) | 90 |
|  | 0.5 mg semaglutide vs GLP-1 RAs | 3 | 413 | 408 | 1.13 (0.90,1.43) | 57 |
|  | 1.0 mg semaglutide vs GLP-1 RAs | 5 | 1100 | 1106 | 1.38 (1.12,1.71) | 88 |
|  | 0.5 mg semaglutide vs DPP-4i | 3 | 800 | 800 | 1.89 (1.53,2.32) | 75 |
|  | 1.0 mg semaglutide vs DPP-4i | 3 | 801 | 800 | 2.15 (1.78,2.59) | 72 |
|  | 1.0 mg semaglutide vs SGLT-2i | 1(canagliflozin) | 394 | 394 | 1.47 (1.29,1.67) | NA |
|  | 1.0 mg semaglutide vs insulinotropic polypeptide–GLP-1 RA | 1(Tirzepatide) | 461 | 461 | 0.95 (0.90, 1.01) | NA |
|  | 0.5 mg semaglutide vs Insulin | 1(Insulin glargine) | 362 | 360 | 1.51 (1.29,1.77) | NA |
|  | 1.0 mg semaglutide vs Insulin | 1(Insulin glargine) | 360 | 360 | 1.93 (1.67,2.23) | NA |
|  | 0.5 mg semaglutide vs additional OADs | 1 | 239 | 120 | 2.01 (1.61,2.50) | NA |
|  | 1.0 mg semaglutide vs additional OADs | 1 | 241 | 120 | 2.18 (1.76,2.71) | NA |
| Number of participants achieving HbA1c <6.5% | 0.5 mg semaglutide vs placebo | 3 | 308 | 308 | 5.02 (3.37,7.48) | 0 |
|  | 1.0 mg semaglutide vs placebo | 4 | 455 | 459 | 8.66 (4.41,17.81) | 66 |
|  | 0.5 mg semaglutide vs active comparator | 7 | 1750 | 1624 | 2.53 (1.63,3.92) | 92 |
|  | 1.0 mg semaglutide vs active comparator | 10 | 2833 | 2715 | 2.93 (2.13,4.03) | 94 |
|  | 0.5 mg semaglutide vs GLP-1 RAs | 2 | 349 | 344 | 0.91 (0.32,2.55) | 86 |
|  | 1.0 mg semaglutide vs GLP-1 RAs | 4 | 1037 | 1041 | 1.78 (1.32,2.40) | 85 |
|  | 0.5 mg semaglutide vs DPP-4i | 3 | 800 | 800 | 4.05 (2.27,7.22) | 85 |
|  | 1.0 mg semaglutide vs DPP-4i | 3 | 801 | 800 | 5.50 (2.73,11.08) | 90 |
|  | 1.0 mg semaglutide vs SGLT-2i | 1(canagliflozin) | 394 | 394 | 2.24 (1.83,2.73) | NA |
|  | 0.5 mg semaglutide vs Insulin | 1(Insulin glargine) | 362 | 360 | 2.13 (1.64,2.77) | NA |
|  | 1.0 mg semaglutide vs Insulin | 1(Insulin glargine) | 360 | 360 | 3.10 (2.43,3.95) | NA |
|  | 0.5 mg semaglutide vs additional OADs | 1 | 239 | 120 | 4.71 (3.06,7.27) | NA |
|  | 1.0 mg semaglutide vs additional OADs | 1 | 241 | 120 | 5.34 (3.47,8.21) | NA |
| Number of participants achieving HbA1c <7.0% without weight gain | 0.5 mg semaglutide vs placebo | 2 | 260 | 262 | 5.01 (2.15,11.67) | 81 |
|  | 1.0 mg semaglutide vs placebo | 3 | 412 | 413 | 5.05 (2.94,8.68) | 77 |
|  | 0.5 mg semaglutide vs active comparator | 6 | 1702 | 1579 | 2.45 (1.85,3.25) | 89 |
|  | 1.0 mg semaglutide vs active comparator | 8 | 2396 | 2271 | 2.61 (1.90,3.58) | 95 |
|  | 0.5 mg semaglutide vs GLP-1 RAs | 1 | 301 | 299 | 1.46 (1.25,1.70) | NA |
|  | 1.0 mg semaglutide vs GLP-1 RAs | 3 | 994 | 991 | 1.73 (1.22,2.45) | 94 |
|  | 0.5 mg semaglutide vs DPP-4i | 3 | 800 | 800 | 2.50 (1.95,3.22) | 69 |
|  | 1.0 mg semaglutide vs DPP-4i | 3 | 801 | 800 | 2.95 (2.31,3.77) | 70 |
|  | 1.0 mg semaglutide vs SGLT-2i | 1(canagliflozin) | 394 | 394 | 2.24 (1.83,2.73) | NA |
|  | 0.5 mg semaglutide vs Insulin | 1(Insulin glargine) | 362 | 360 | 2.13 (1.64,2.77) | NA |
|  | 1.0 mg semaglutide vs Insulin | 1(Insulin glargine) | 360 | 360 | 3.10 (2.43,3.95) | NA |
|  | 0.5 mg semaglutide vs additional OADs | 1 | 239 | 120 | 4.17 (3.06,7.27) | NA |
|  | 1.0 mg semaglutide vs additional OADs | 1 | 241 | 120 | 5.34 (3.47,8.21) | NA |
| Participants achieving bodyweight reduction≥5% | 0.5 mg semaglutide vs placebo | 4 | 372 | 437 | 2.72 (1.47,5.02) | 68 |
|  | 1.0 mg semaglutide vs placebo | 6 | 586 | 657 | 5.28 (4.14,6.75) | 0 |
|  | 0.5 mg semaglutide vs active comparator | 8 | 1814 | 1688 | 2.92 (1.85,4.60) | 87 |
|  | 1.0 mg semaglutide vs active comparator | 11 | 2896 | 2780 | 3.60 (2.26,5.72) | 96 |
|  | 0.5 mg semaglutide vs GLP-1 RAs | 3 | 413 | 408 | 1.34 (0.76,2.37) | 62 |
|  | 1.0 mg semaglutide vs GLP-1 RAs | 5 | 1100 | 1106 | 2.33 (1.65,3.30) | 85 |
|  | 0.5 mg semaglutide vs DPP-4i | 3 | 800 | 800 | 3.90 (2.09,7.28) | 81 |
|  | 1.0 mg semaglutide vs DPP-4i | 3 | 801 | 800 | 6.25 (2.83,13.81) | 89 |
|  | 1.0 mg semaglutide vs SGLT-2i | 1(canagliflozin) | 394 | 394 | 1.10 (0.96,1.27) | NA |
|  | 0.5 mg semaglutide vs Insulin | 1(Insulin glargine) | 362 | 360 | 7.84 (4.84,12.71) | NA |
|  | 1.0 mg semaglutide vs Insulin | 1(Insulin glargine) | 360 | 360 | 10.76 (6.69,17.31) | NA |
|  | 0.5 mg semaglutide vs additional OADs | 1 | 239 | 120 | 4.52 (2.14,9.56) | NA |
|  | 1.0 mg semaglutide vs additional OADs | 1 | 241 | 120 | 7.97 (3.83,16.56) | NA |
| Participants achieving bodyweight reduction≥10% | 0.5 mg semaglutide vs placebo | 3 | 308 | 308 | 3.17 (1.38,7.28) | 0 |
|  | 1.0 mg semaglutide vs placebo | 5 | 523 | 528 | 9.08 (4.86,16.96) | 0 |
|  | 0.5 mg semaglutide vs active comparator | 6 | 1750 | 1624 | 4.93 (3.36,7.22) | 0 |
|  | 1.0 mg semaglutide vs active comparator | 10 | 2543 | 2425 | 5.90 (3.74,9.32) | 71 |
|  | 0.5 mg semaglutide vs GLP-1 RAs | 2 | 349 | 344 | 4.27 (2.19,8.34) | NA |
|  | 1.0 mg semaglutide vs GLP-1 RAs | 4 | 1037 | 1041 | 4.25 (3.19,5.67) | 0 |
|  | 0.5 mg semaglutide vs DPP-4i | 3 | 800 | 800 | 7.73 (2.39,25.02) | 38 |
|  | 1.0 mg semaglutide vs DPP-4i | 3 | 801 | 800 | 17.24 (4.29,69.23) | 53 |
|  | 1.0 mg semaglutide vs SGLT-2i | 1(canagliflozin) | 394 | 394 | 2.51 (1.74,3.63) | NA |
|  | 0.5 mg semaglutide vs Insulin | 1(Insulin glargine) | 362 | 360 | 4.64 (1.95,11.07) | NA |
|  | 1.0 mg semaglutide vs Insulin | 1(Insulin glargine) | 360 | 360 | 9.50 (4.15,21.75) | NA |
|  | 0.5 mg semaglutide vs additional OADs | 1 | 239 | 120 | 10.04 (1.36,73.93) | NA |
|  | 1.0 mg semaglutide vs additional OADs | 1 | 241 | 120 | 20.91 (2.91,150.11) | NA |
| AEs | 0.5 mg semaglutide vs placebo | 5 | 1198 | 1261 | 1.13 (0.94,1.36) | 83 |
|  | 1.0 mg semaglutide vs placebo | 7 | 1408 | 1484 | 1.11 (1.00,1.24) | 67 |
|  | 0.5 mg semaglutide vs active comparator | 8 | 1813 | 1688 | 1.08 (1.03,1.14) | 11 |
|  | 1.0 mg semaglutide vs active comparator | 11 | 2895 | 2780 | 1.05 (1.00,1.10) | 51 |
|  | 0.5 mg semaglutide vs GLP-1 RAs | 3 | 413 | 408 | 1.24 (0.93,1.65) | 48 |
|  | 1.0 mg semaglutide vs GLP-1 RAs | 5 | 1099 | 1106 | 0.97 (0.80,1.17) | 20 |
|  | 0.5 mg semaglutide vs DPP-4i | 3 | 799 | 800 | 1.23 (0.99,1.53) | 0 |
|  | 1.0 mg semaglutide vs DPP-4i | 3 | 801 | 800 | 1.14 (0.92,1.41) | 0 |
|  | 1.0 mg semaglutide vs SGLT-2i | 1(canagliflozin) | 394 | 394 | 1.06 (0.97,1.15) | NA |
|  | 1.0 mg semaglutide vs insulinotropic polypeptide–GLP-1 RA | 1(Tirzepatide) | 469 | 470 | 1.01 (0.92, 1.11) | NA |
|  | 0.5 mg semaglutide vs Insulin | 1(Insulin glargine) | 362 | 360 | 1.23 (0.90,1.69) | NA |
|  | 1.0 mg semaglutide vs Insulin | 1(Insulin glargine) | 360 | 360 | 1.46 (1.06,2.01) | NA |
|  | 0.5 mg semaglutide vs additional OADs | 1 | 239 | 120 | 2.47 (1.44,4.24) | NA |
|  | 1.0 mg semaglutide vs additional OADs | 1 | 241 | 120 | 2.89 (1.66,5.04) | NA |
| SAEs | 0.5 mg semaglutide vs placebo | 5 | 1198 | 1261 | 0.90 (0.80,1.02) | 19 |
|  | 1.0 mg semaglutide vs placebo | 7 | 1408 | 1484 | 0.95 (0.84,1.08) | 0 |
|  | 0.5 mg semaglutide vs active comparator | 8 | 1813 | 1688 | 1.17 (0.90,1.52) | 13 |
|  | 1.0 mg semaglutide vs active comparator | 11 | 2895 | 2780 | 1.04 (0.85,1.28) | 0 |
|  | 0.5 mg semaglutide vs GLP-1 RAs | 3 | 413 | 408 | 1.98 (0.35,11.19) | 62 |
|  | 1.0 mg semaglutide vs GLP-1 RAs | 5 | 1099 | 1106 | 1.05 (0.68,1.64) | 32 |
|  | 0.5 mg semaglutide vs DPP-4i | 3 | 799 | 800 | 1.28 (0.84,1.93) | 2 |
|  | 1.0 mg semaglutide vs DPP-4i | 3 | 801 | 800 | 1.17 (0.77,1.78) | 0 |
|  | 1.0 mg semaglutide vs SGLT-2i | 1(canagliflozin) | 394 | 394 | 0.86 (0.46,1.58) | NA |
|  | 0.5 mg semaglutide vs Insulin | 1(Insulin glargine) | 362 | 360 | 1.23 (0.65,2.33) | NA |
|  | 1.0 mg semaglutide vs Insulin | 1(Insulin glargine) | 360 | 360 | 0.94 (0.48,1.86) | NA |
|  | 0.5 mg semaglutide vs additional OADs | 1 | 239 | 120 | 1.21 (0.51,2.85) | NA |
|  | 1.0 mg semaglutide vs additional OADs | 1 | 241 | 120 | 0.73 (0.29,1.85) | NA |
| Severe or BG-confirmed hypoglycaemia | 0.5 mg semaglutide vs placebo | 4 | 1150 | 1251 | 1.08 (0.91,1.29) | 0 |
|  | 1.0 mg semaglutide vs placebo | 6 | 1365 | 1438 | 1.09 (0.91,1.30) | 18 |
|  | 0.5 mg semaglutide vs active comparator | 7 | 1765 | 1643 | 0.56 (0.36,0.88) | 0 |
|  | 1.0 mg semaglutide vs active comparator | 10 | 2852 | 2730 | 0.81 (0.60,1.09) | 0 |
|  | 0.5 mg semaglutide vs GLP-1 RAs | 2 | 365 | 363 | 0.98 (0.23,4.16) | 0 |
|  | 1.0 mg semaglutide vs GLP-1 RAs | 4 | 1056 | 1056 | 0.94 (0.61,1.43) | 0 |
|  | 0.5 mg semaglutide vs DPP-4i | 3 | 799 | 800 | 1.00 (0.39,2.54) | 0 |
|  | 1.0 mg semaglutide vs DPP-4i | 3 | 801 | 800 | 1.00 (0.40,2.47) | 4 |
|  | 1.0 mg semaglutide vs SGLT-2i | 1(canagliflozin) | 394 | 394 | 1.20 (0.37,3.90) | NA |
|  | 0.5 mg semaglutide vs Insulin | 1(Insulin glargine) | 362 | 360 | 0.39 (0.21,0.72) | NA |
|  | 1.0 mg semaglutide vs Insulin | 1(Insulin glargine) | 360 | 360 | 0.50 (0.28,0.87) | NA |
|  | 0.5 mg semaglutide vs additional OADs | 1 | 239 | 120 | 0.75 (0.12,4.55) | NA |
|  | 1.0 mg semaglutide vs additional OADs | 1 | 241 | 120 | 1.51 (0.30,7.58) | NA |
| nausea | 0.5 mg semaglutide vs placebo | 5 | 1198 | 1261 | 2.52 (1.98,3.19) | 0 |
|  | 1.0 mg semaglutide vs placebo | 7 | 1408 | 1484 | 3.36 (2.71,4.17) | 39 |
|  | 0.5 mg semaglutide vs active comparator | 8 | 1813 | 1688 | 2.77 (1.67,4.59) | 75 |
|  | 1.0 mg semaglutide vs active comparator | 11 | 2895 | 2780 | 2.77 (1.78,4.31) | 86 |
|  | 0.5 mg semaglutide vs GLP-1 RAs | 3 | 413 | 408 | 1.76 (1.22,2.54) | 0 |
|  | 1.0 mg semaglutide vs GLP-1 RAs | 5 | 1099 | 1106 | 1.65 (1.04,2.62) | 72 |
|  | 0.5 mg semaglutide vs DPP-4i | 3 | 799 | 800 | 3.74 (1.80,7.76) | 39 |
|  | 1.0 mg semaglutide vs DPP-4i | 3 | 801 | 800 | 5.92 (1.78,19.75) | 74 |
|  | 1.0 mg semaglutide vs SGLT-2i | 1(canagliflozin) | 394 | 394 | 3.42 (2.26,5.18) | NA |
|  | 0.5 mg semaglutide vs Insulin | 1(Insulin glargine) | 362 | 360 | 7.21 (3.93,13.25) | NA |
|  | 1.0 mg semaglutide vs Insulin | 1(Insulin glargine) | 360 | 360 | 7.63 (4.16,13.99) | NA |
|  | 0.5 mg semaglutide vs additional OADs | 1 | 239 | 120 | 16.43 (2.21,122.17) | NA |
|  | 1.0 mg semaglutide vs additional OADs | 1 | 241 | 120 | 28.07 (3.82,206.23) | NA |
| diarrhoea | 0.5 mg semaglutide vs placebo | 5 | 1198 | 1261 | 2.04 (1.08,3.87) | 52 |
|  | 1.0 mg semaglutide vs placebo | 7 | 1408 | 1484 | 1.88 (1.42,2.49) | 11 |
|  | 0.5 mg semaglutide vs active comparator | 8 | 1813 | 1688 | 2.28 (1.78,2.91) | 14 |
|  | 1.0 mg semaglutide vs active comparator | 11 | 2895 | 2780 | 1.72 (1.26,2.35) | 73 |
|  | 0.5 mg semaglutide vs GLP-1 RAs | 3 | 413 | 408 | 2.02 (1.27,3.22) | 0 |
|  | 1.0 mg semaglutide vs GLP-1 RAs | 5 | 1099 | 1106 | 1.12 (0.87,1.44) | 24 |
|  | 0.5 mg semaglutide vs DPP-4i | 3 | 799 | 800 | 2.59 (1.83,3.65) | 18 |
|  | 1.0 mg semaglutide vs DPP-4i | 3 | 801 | 800 | 2.37 (1.67,3.35) | 0 |
|  | 1.0 mg semaglutide vs SGLT-2i | 1(canagliflozin) | 394 | 394 | 1.62 (1.10,2.38) | NA |
|  | 0.5 mg semaglutide vs Insulin | 1(Insulin glargine) | 362 | 360 | 4.19 (2.36,7.43) | NA |
|  | 1.0 mg semaglutide vs Insulin | 1(Insulin glargine) | 360 | 360 | 5.10 (2.90,8.98) | NA |
|  | 0.5 mg semaglutide vs additional OADs | 1 | 239 | 120 | 1.56 (0.68,3.59) | NA |
|  | 1.0 mg semaglutide vs additional OADs | 1 | 241 | 120 | 2.62 (1.18,5.81) | NA |
| vomiting | 0.5 mg semaglutide vs placebo | 5 | 1198 | 1261 | 2.21 (1.61,3.02) | 0 |
|  | 1.0 mg semaglutide vs placebo | 7 | 1408 | 1484 | 3.80 (2.80,5.15) | 0 |
|  | 0.5 mg semaglutide vs active comparator | 8 | 1813 | 1688 | 2.70 (1.93,3.77) | 0 |
|  | 1.0 mg semaglutide vs active comparator | 11 | 2895 | 2780 | 2.23 (1.45,3.46) | 71 |
|  | 0.5 mg semaglutide vs GLP-1 RAs | 3 | 413 | 408 | 2.74 (1.54,4.89) | 0 |
|  | 1.0 mg semaglutide vs GLP-1 RAs | 5 | 1099 | 1106 | 1.26 (0.94,1.69) | 0 |
|  | 0.5 mg semaglutide vs DPP-4i | 3 | 799 | 800 | 3.58 (1.99,6.43) | 0 |
|  | 1.0 mg semaglutide vs DPP-4i | 3 | 801 | 800 | 4.42 (2.49,7.85) | 0 |
|  | 1.0 mg semaglutide vs SGLT-2i | 1(canagliflozin) | 394 | 394 | 5.56 (2.77,11.14) | NA |
|  | 0.5 mg semaglutide vs Insulin | 1(Insulin glargine) | 362 | 360 | 2.25 (1.09,4.67) | NA |
|  | 1.0 mg semaglutide vs Insulin | 1(Insulin glargine) | 360 | 360 | 3.63 (1.82,7.25) | NA |
|  | 0.5 mg semaglutide vs additional OADs | 1 | 239 | 120 | 3.39 (0.75,15.29) | NA |
|  | 1.0 mg semaglutide vs additional OADs | 1 | 241 | 120 | 3.64 (0.81,16.28) | NA |
| Acute pancreatitis | 0.5mg semaglutide vs placebo | 5 | 1198 | 1261 | 2.00 (0.50, 7.95) | NA |
|  | 1.0mg semaglutide vs placebo | 7 | 1408 | 1484 | 0.48 (0.16, 1.46) | 38 |
|  | 0.5mg semaglutide vs active comparator | 8 | 1813 | 1688 | 5.97 (0.72, 49.46) | 0 |
|  | 1.0mg semaglutide vs active comparator | 10 | 2501 | 2386 | 0.67 (0.19, 2.35) | 0 |
|  | 0.5mg semaglutide vs GLP-1 RAs | 3 | 413 | 408 | 0.71 (0.14, 3.62) | 0 |
|  | 1.0mg semaglutide vs GLP-1 RAs | 5 | 1099 | 1106 | 0.45 (0.10, 2.02) | 0 |
|  | 0.5mg semaglutide vs DPP4i | 3 | 799 | 800 | 7.02 (0.36,136.28) | NA |
|  | 1.0mg semaglutide vs DPP4i | 3 | 801 | 800 | 3.01 (0.12,74.20) | NA |
|  | 0.5mg semaglutide vs Insulin | 1(Insulin glargine) | 362 | 360 | 5.00 (0.24,104.51) | NA |
|  | 1.0mg semaglutide vs Insulin | 1(Insulin glargine) | 360 | 360 | NE | NA |
|  | 0.5mg semaglutide vs additional OADs | 1 | 239 | 120 | NE | NA |
|  | 1.0mg semaglutide vs additional OADs | 1 | 241 | 120 | NE | NA |
| Diabetic retinopathy | 0.5 mg semaglutide vs placebo | 4 | 1070 | 1132 | 3.40 (0.83,13.88) | 0 |
|  | 1.0 mg semaglutide vs placebo | 5 | 1209 | 1284 | 0.67 (0.29,1.56) | 16 |
|  | 0.5 mg semaglutide vs active comparator | 8 | 1813 | 1688 | 1.22 (0.76,1.98) | 0 |
|  | 1.0 mg semaglutide vs active comparator | 10 | 2491 | 2375 | 0.86 (0.58,1.28) | 0 |
|  | 0.5 mg semaglutide vs GLP-1 RAs | 3 | 413 | 408 | 0.71 (0.14,3.62) | 0 |
|  | 1.0 mg semaglutide vs GLP-1 RAs | 4 | 695 | 701 | 0.65 (0.25,1.70) | 0 |
|  | 0.5 mg semaglutide vs DPP-4i | 3 | 799 | 800 | 1.44 (0.77,2.72) | 21 |
|  | 1.0 mg semaglutide vs DPP-4i | 3 | 801 | 800 | 0.94 (0.48,1.87) | 35 |
|  | 1.0 mg semaglutide vs SGLT-2i | 1(canagliflozin) | 394 | 394 | 0.59 (0.26,1.37) | NA |
|  | 0.5 mg semaglutide vs Insulin | 1(Insulin glargine) | 362 | 360 | 2.99 (0.12,73.68) | NA |
|  | 1.0 mg semaglutide vs Insulin | 1(Insulin glargine) | 360 | 360 | 3.01 (0.12,74.09) | NA |
|  | 0.5 mg semaglutide vs additional OADs | 1 | 239 | 120 | 0.92 (0.33,2.54) | NA |
|  | 1.0 mg semaglutide vs additional OADs | 1 | 241 | 120 | 1.35 (0.51,3.55) | NA |
